# Salivary biomarkers in oral cancer diagnosis: advancing conventional treatment strategies

**DOI:** 10.1016/j.mmr.2026.100018

**Published:** 2026-04-17

**Authors:** Ankit Paul, Tarun Kumar Upadhyay, Adil Ali, Hemant Singh, Mohd Saeed, Farrukh Aqil

**Affiliations:** a Department of Biotechnology, Parul Institute of Applied Sciences and Research and Development Cell, Parul University, Vadodara 391760, Gujarat, India; b Biological Sciences, Khalifa University of Science and Technology, Abu Dhabi 127788, United Arab Emirates; c Center for Biotechnology, Khalifa University of Science and Technology, Abu Dhabi 127788, United Arab Emirates; d Department of Biology, College of Science, University of Hail, PO BOX 2240, Hail, Saudi Arabia; e Department of Medicine, Brown Cancer Center, University of Louisville, Louisville, KY, 40202, USA

**Keywords:** Oral cancer, Oral squamous cell carcinoma (OSCC), Salivary biomarker, Liquid biopsy, Early detection

## Abstract

Oral squamous cell carcinoma (OSCC) is a major global health challenge, with most cases being diagnosed at advanced stages. Traditional diagnostic methods are often invasive and costly, and can delay diagnosis. Saliva has emerged as a promising non-invasive source of biomarkers for OSCC detection. This highlights the need for accessible, non-invasive, and sensitive biomarkers for OSCC detection. This review critically evaluates the current status and future potential of salivary biomarkers in OSCC, with an emphasis on their diagnostic efficacy, sensitivity, specificity, clinical validation, and advantages over traditional serum- and plasma-based markers. Saliva is a promising liquid for biopsy due to its non-invasive collection and molecular richness. We summarize evidence on diverse salivary biomarkers, including microRNAs (miRNAs), proteins, metabolites, circulating tumor cells (CTCs), and circulating tumor DNA (ctDNA), highlighting their dysregulation in OSCC and diagnostic utility. Particular emphasis is placed on CTCs, ctDNA, and miRNAs, which demonstrate stability in saliva and potential for early detection. We further discuss advances in next-generation sequencing, mass spectrometry, and artificial intelligence/machine learning that enable the development of biomarker panels with improved diagnostic accuracy over single markers. Despite challenges such as sample heterogeneity and the lack of standardized protocols, salivary biomarkers hold strong potential to transform OSCC care by enabling earlier detection, guiding personalized therapies, and supporting non-invasive disease monitoring. However, achieving methodological standardization, validating biomarkers across diverse cohorts, and integrating them into clinical workflows are imperative before their routine application in practice.

## 1 Background

Oral cancers (OC) include malignant growths originating in the mucosal epithelium of the mouth, larynx, oropharynx, nasopharynx, and other adjacent oral structures [1]. Clinically, “oral cancer” is often grouped under the broader category of “head and neck” squamous cell carcinoma (HNSCC) to improve in-depth knowledge of cancers affecting this anatomical area, thereby enhancing diagnostic accuracy and treatment outcomes [2]. Among the various histological types, SCC is by far the most predominant, with a well-established association with etiological factors such as tobacco and alcohol abuse [2], [3]. Other, less common forms include non-squamous malignancies and a small number of rare or non-malignant neoplasms.

Globally, oral squamous cell carcinoma (OSCC) ranks as the 6th most prevalent cancer, with a particularly high incidence in developing countries according to the WHO GLOBOCAN 2022 statistics [4]. Cancers associated with the lip and oral cavity account for approximately 0.3 million new cases and approximately 0.188 million deaths annually, ranking 16th worldwide [4]. Other subsites include the nasopharynx (23rd; 0.120 million cases, 0.073 million deaths), oropharynx (24th; 0.100 million cases, 0.052 million deaths), hypopharynx (25th; 0.086 million cases, 0.041 million deaths), and salivary glands (28th; 0.055 million cases, 0.024 million deaths) [4]. The burden is especially severe in regions where tobacco is consumed in both smoking and chewing forms, often in conjunction with alcohol. The incidence and mortality rates vary widely across geographic, demographic, and cultural groups, reflecting differences in exposure to risk factors, socioeconomic status, and access to healthcare [5], [6]. Each year, more than 400,000 new OSCC cases are reported worldwide, with nearly two-thirds arising in South Asian nations, including India, Bangladesh, Sri Lanka, Indonesia, and Pakistan [7], [8]. In these high-risk regions, OSCC accounts for more than 25% of all cancers diagnosed annually. The disease is strongly age-dependent, with the highest prevalence in individuals over 60 years; however, the incidence among patients younger than 40 years has been increasing regardless of sex [7], [9].

Despite therapeutic advances, the prognosis of OSCC remains poor because of late-stage diagnosis, high tumor recurrence, and drug resistance development [8], [10]. Many patients seek medical attention only after symptoms emerge, such as persistent soreness, bleeding, or visible lesions, indicating a late OC stage neoplasm [11], [12], [13], [14], [15]. Simultaneously, professional factors (such as misdiagnosis or inappropriate initial diagnostic management) also contribute to these delays [15], [16], [17], [18]. These delays contribute to a mere 50% overall 5-year survival rate [14], [15], [16], whereas early stages I/II detection allows 80% patient survivability [7], [8], [10]. Unfortunately, over half of all OSCC patients are still diagnosed at advanced stages (III and IV), largely due to the absence of early clinical signs [8], [10]. Prognosis worsens as the disease progresses, and tumors become anatomically more difficult to access. For example, lip cancers generally have better outcomes than oropharyngeal tumors do [8], [12]. Importantly, the clinical stage at diagnosis remains the most critical determinant of prognosis [12]. The persistently high mortality of OSCC underscores the urgent need for earlier and more accurate diagnoses [19], [20]. Early-stage detection not only improves survival but also reduces treatment-related morbidity, highlighting the importance of both public awareness and clinical vigilance [19]. Consequently, there is a growing emphasis among researchers and clinicians on developing innovative and accessible diagnostic tools and screening methods to facilitate earlier detection of oral cancer [14], [17].

OSCC is closely associated with the oral microenvironment, where it interacts directly with saliva, a complex biological fluid secreted by the salivary glands [20]. Saliva, often termed “the mirror of the body”, has gained recognition as a promising diagnostic medium [19]. Liquid biopsy using saliva offers a unique opportunity for non-invasive cancer detection, longitudinal disease monitoring, and assessment of therapeutic response [20]. The advantages of saliva sampling include 1) reflecting both local and systemic changes; 2) rapid, simple, and accessible collection; 3) the ability to obtain larger volumes for repeated testing; and 4) the presence of diverse analytes, including circulating tumor DNA (ctDNA), microRNAs (miRNAs), circulating tumor cells (CTCs) microbial signatures, proteins, and metabolites [19], [20].

Recent studies have emphasized saliva’s role as a reservoir of OSCC-specific biomarkers, positioning it as an attractive diagnostic alternative to conventional tissue- or blood-based methods [19], [20]. Nonetheless, several challenges hinder translation into clinical practice. These include variability in sample collection and processing, heterogeneity among patient cohorts, lack of standardized protocols, and limited large-scale clinical validation [19]. Moreover, the integration of advanced technologies such as next-generation sequencing, mass spectrometry, and artificial intelligence into routine clinical diagnostics remains in its infancy.

**Fig. 1:**
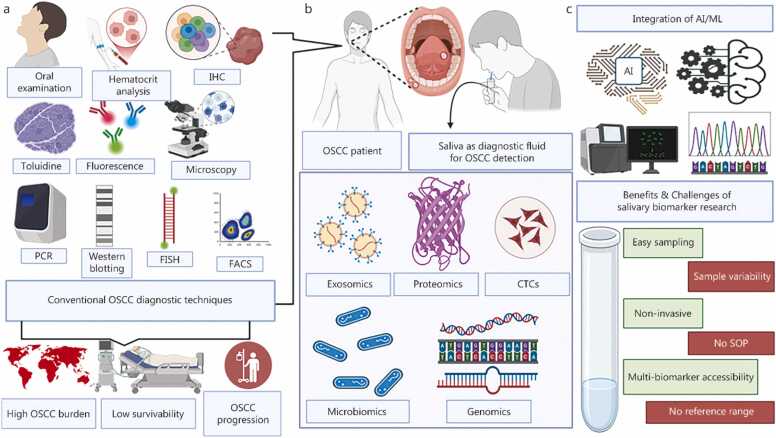
Saliva-based biomarker landscape for OSCC: from conventional diagnostic techniques to multi-omics and AI-integrated detection. This figure illustrates the emerging landscape of OSCC diagnosis, delineating the shift from conventional diagnostic techniques to a salivary-based diagnostic approach integrated with artificial intelligence and machine learning. **a** Represents conventional techniques (such as oral examination, hematocrit analysis, IHC, toluidine staining, fluorescence-based ELISA techniques, microscopic evaluation, PCR, western blotting, FISH, FACS) that failed to reduce the OSCC burden and resulted in late-stage detection. **b,c** While saliva-based diagnosis (**b**) holds the advantage of non-invasiveness, being pain-free, and allows prompt OSCC detection through easy sampling and multi-panel biomarkers. When integrated with AI/ML (**c**), it can also predict the disease risk, but it has certain disadvantages, such as sample variability, no SOPs, and no reference range. Alleviating these disadvantages can lead to benchside detection. OSCC. Oral squamous cell carcinoma; IHC. Immunohistochemistry; PCR. Polymerase chain reaction; FISH. Fluorescence in situ hybridization; FACS. Fluorescence-assisted cell sorting; CTCs. Circulating tumor cells; AI. Artificial intelligence; ML. Machine learning; SOP. Standard operating procedure.

This review provides an updated overview of OSCC epidemiology, established and emerging risk factors, and current diagnostic limitations. Saliva is a promising, non-invasive diagnostic medium for biomarker discovery, with the potential to enable early detection, personalized therapy, and continuous disease monitoring. By identifying key research gaps, such as inadequate standardization, limited databases, and insufficient clinical validation, this review underscores the need for coordinated efforts to advance saliva-based diagnostics and ultimately reduce the global OSCC burden, as shown in Fig. 1.

## 2 Etiological factors of OSCC

**Fig. 2:**
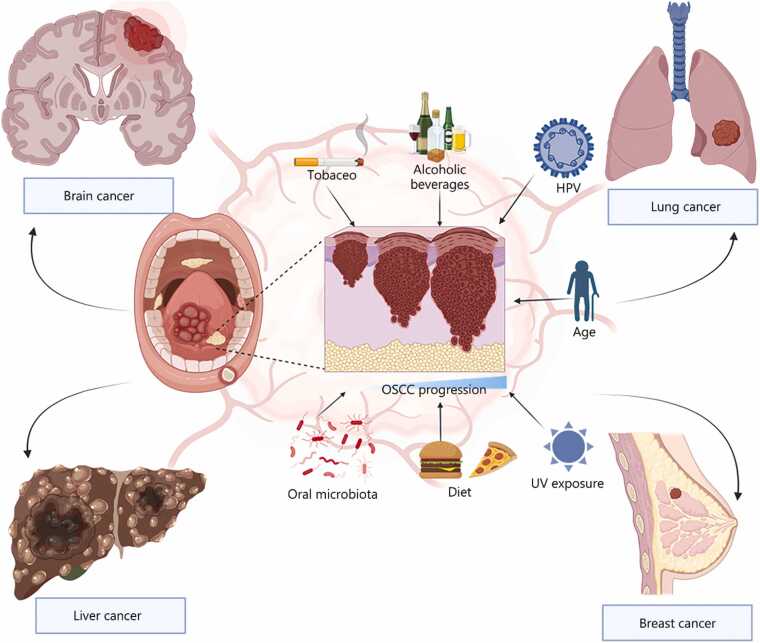
Etiological factors, tumor advancement, and systemic cancer associations of OSCC. Etiological factors of OSCC. Major risk factors include tobacco uptake (either smoking or chewing), alcohol abuse, HPV infection, oral microbiota, age, sex, and exposure to UV, which cause dysphagia and ulceration, which are early signs of OSCC. Under chronic exposure, these risk factors contribute to OSCC development and advanced-stage OSCC metastasis to different organs if not detected early. OSCC. Oral squamous cell carcinoma; HPV. Human papillomavirus; UV. Ultraviolet.

Several risk factors have been identified as contributing to, or implicated in, OSCC in both the etiology and progression of the disease; the most recognized among these are chemicals, notably tobacco and alcohol. Human papillomavirus (HPV), syphilis, and chronic infections such as candidiasis have also been associated with OSCC. Factors related to oral health, such as poor oro-dental hygiene, along with nutritional deficiencies and alterations in the oral microbiota, have also been found to significantly influence disease pathogenicity and advancement [5], as shown in Fig. 2**.**

### 2.1 Tobacco abuse and nicotine dependency

Tobacco use and nicotine addiction remain the most significant etiological factors for the progression of OSCC worldwide [21]. The act of smoking through cigarettes, cigars, or pipes exposes oral tissues to numerous carcinogenic compounds. These substances directly damage the mucosal lining, triggering cellular changes that can lead to cancer initiation [21], [22]. In addition to smoking, the consumption of smokeless tobacco-based products such as chewing tobacco, snuff, and betel quid (a mixture of areca nut with or without tobacco) is especially prevalent in Southeast Asian populations [22]. These commodities are rich in oncogenic nitrosamine derivatives and alkaloids, which contribute to persistent mucosal inflammation, the formation of precancerous lesions such as leucoplakia, and, over time, malignant transformation [21]. The strong correlation between tobacco exposure and oral carcinogenesis is highlighted by the fact that smokers are more prone to OSCC risk than non-smokers are, according to epidemiological studies [22], [23].

### 2.2 Alcohol consumption

Alcohol abuse is considered the chief etiological factor for the development of OSCC, especially when alcohol is used in conjunction with tobacco [23]. There is also a synergistic influence between tobacco and alcohol use, which greatly increases the likelihood of cancer [23], [24]. Alcohol allows for greater absorptivity of the buccal mucosa and the absorption of carcinogenic compounds found in tobacco or other related products [23]. Individuals who consume more than 3 – 4 alcoholic beverages per day have the highest probability of developing OSCC, especially when this habit is accompanied by smoking [24]. Additionally, alcohol contributes to carcinogenesis by impairing DNA repair processes and promoting persistent inflammation within the oral epithelium, which are known to facilitate the formation of malignant lesions [23], [25].

### 2.3 Betel-quid and areca nut chewing

Betel-quid (BQ) and areca nut abuse, a longstanding ethanographic custom in Southeast Asian nations, is a well-established etiological factor for OSCC [26]. BQ typically consists of catechu, pickling lime, and areca nuts, and is often consumed with or without the addition of tobacco. The International Agency for Research on Cancer (IARC) has vilified areca nut as a Group 1 carcinogen, highlighting its strong carcinogenic potential. Chronic use of areca nuts is associated with fibrotic alterations in the buccal mucosa, accelerating the development of oral submucous fibrosis (OSF), a clinically recognized preneoplastic condition [27]. OSF patients are at a substantially increased risk of developing OSCC [23], [27].

### 2.4 HPV infection

HPV is a critical etiological factor and is integral to the pathogenesis of OSCC, particularly oropharyngeal cancer involving the tongue, basals and tonsils, with its oncogenic potential originating from a distinct infection pathway coupled with complex molecular mechanisms that subvert host defenses and cellular signaling [23]. The infection process typically involves microabrasions in the oral mucosa, which expose basal epithelial cells, the primary targets for HPV entry [28]. Once HPV virions gain access to these cells, they attach to heparan sulfate proteoglycans and enter through clathrin-mediated endocytosis, subsequently positioning their genome in the nucleus as episomes. This initial asymptomatic infection permits the virus to persist while evading immune recognition, particularly as it avoids viremia and systemic dispersal, remaining localized within epithelial tissues [23], [28].

At the molecular level, HPV-driven oncogenesis in OSCC is predominantly facilitated by the viral oncoproteins *E6* and *E7*, which disrupt the functions of host tumor suppressors [28]. *E6* accelerates the ubiquitin-mediated degradation of p53, thereby impairing the DNA damage response, apoptosis, and cell cycle regulation. Concurrently, *E7* interacts with the retinoblastoma protein (pRb), resulting in its inactivation and the subsequent release of Early region 2 binding factor (E2F) transcription factors that drive uncontrolled entry into the S phase and cellular proliferation [23], [28]. These interactions are central to the ability of HPV to hijack cell cycle control and induce genomic instability, which ultimately leads to malignant transformation.

The pathogenesis of HPV-mediated OSCC involves multifaceted interactions among viral persistence, immune evasion, and altered host signaling pathways [23]. By downregulating antigen-presenting mechanisms, such as major histocompatibility complex (MHC) class I expression, and interfering with interferon signaling, HPV-infected cells are able to evade immune clearance, allowing for the accumulation of oncogenic mutations [28], [29]. The integration of viral DNA into the host genome, which often disrupts the viral *E2* gene, further deregulates the expression of *E6* and *E7*, thus cementing their oncogenic influence and facilitating the clonal expansion of transformed epithelial cells. Clinically, this results in distinct subgroups of OSCC, in which HPV-positive tumors are frequently associated with a more favorable prognosis than are HPV-negative tumors, despite their dependence on potent viral oncogenic drivers [30].

HPV also modifies several critical signaling pathways that contribute to the progression of OSCC. Activation of the phosphatidylinositol 3-kinase/protein kinase B/mammalian target of rapamycin (PI3K/Akt/mTOR) pathway, which promotes cell survival, metabolic reprogramming, and resistance to apoptosis, is frequently observed [28], [29]. Additionally, HPV influences the Wnt/β-catenin pathway, enhancing both proliferative and invasive potential, while modulating Notch signaling, which is essential for epithelial differentiation [29]. Furthermore, *E6*-mediated activation of telomerase (hTERT) supports limitless replication, and *E7*-driven chromosomal instability contributes to tumor heterogeneity [28]. Collectively, these altered signaling cascades, in conjunction with the hallmark inactivation of p53 and Rb, provide the molecular framework through which HPV drives the onset and progression of OSCC [29].

### 2.5 Diet and nutrition

Dietary and nutritional factors are increasingly recognized as important contributors to the risk of developing oral neoplasms [31]. Diets deficient in fruits and vegetables, key sources of antioxidants, vitamins A, C, and E, and dietary fiber, have been associated with increased susceptibility to oral malignancies [31], [32]. In contrast, the frequent consumption of certain foods, particularly processed red meats and brined fish, has been correlated with an increased risk of neoplastic development [23], [33].

### 2.6 Other emerging risk factors

In addition to established risk factors, several other contributors have been recognized in the advancement of OSCC neoplasms. These include poor oral hygiene, persistent mechanical inflammation caused by misfitting dentures or misaligned dentition, and environmental exposure to carcinogens such as asbestos and other heavy metal elements [32], [34]. Genetic susceptibility, including cancer heredity, may further increase an individual’s vulnerability to developing oral malignancies [23], [34]. Recent clinical and scientific investigations have increasingly focused on the potential oncogenic influence of the oral microbiota, chronic buccal mucosal soreness, and repeated trauma to the oral mucosa from teeth or dental prostheses, highlighting their relevance in the complex etiology of oral malignancy [9], [20].

## 3 Clinical and histopathological presentation of OSCC

OSCC diagnosis is complex due to pronounced biological heterogeneity (exhibiting varying degrees of dysplasia upon oral examination), field cancerization (true margins are difficult to identify because normal-appearing oral mucosal cells may harbour molecular alterations), and phenotypic plasticity (as tumor cells often alter their morphology without any extracellular surface modifications). These challenges are further compounded by anatomical diversity (as epithelial thickness, keratinization, vascular, and lymphatic densities varying extensively in different parts of mouth) and by limited accessibility of oral subsites (such as retromolar trigone, floor of mouth, and posterior region of tongue), particularly in advanced tumors, which mimic benign tumorigenic characteristics which lead to misdiagnosis by general practitioners. However, nearly four decades of accumulated data have enabled detailed analysis of specific subsites, providing a unique opportunity to examine large case series. Therefore, a definitive diagnosis must always be confirmed by biopsy and histopathological evaluation, as clinical presentation alone is often insufficient [35].

OSCC frequently arises from persistent oral potentially malignant disorders (OPMDs), which present as progressive epithelial dysplasia of varying histological grades. While these lesions are established precursors to OSCC, some tumors can also develop de novo from histologically normal oral mucosa [36]. OPMDs exhibit heterogeneous clinical characteristics where several visible mucosal alterations precede malignant transformation, but only a few transform into invasive carcinoma. Some mucosal lesions may remain dormant for prolonged periods, whereas other subsets may advance rapidly [36]. Mucosal alterations in OPMDs include various lesion types, such as oral leukoplakia (OLP), which often appears as a white mucosal patch that can range from mild to severe dysplasia, erythroplakia, nicotine stomatitis, and oral submucous fibrosis (OSF) [37]; Erythroplakia represents red coloration in the mucosa, however it is less prevalent but still holds clinical significance due to a greater risk of *in situ* carcinoma transformation [38]; parallely OSF exhibits burning sensations and stiffening in the oral mucosa due to fibroelastic tissue changes which contributes to restricted mouth opening [39].

Thorough clinical examination remains essential, with particular attention to high-risk sites such as the lateral tongue border and the floor of the mouth. Examination of cervicofacial lymph nodes is equally important, as enlargement may indicate regional metastasis [40]. The size of OSCC lesions varies considerably, ranging from a few millimeters in early disease to several centimeters in advanced stages. Early lesions are typically small and asymptomatic, which often contributes to diagnostic delays. A persistent oral lesion lasting longer than three weeks should lead to suspicion of OSCC. At this stage, lesions often present as erythroleukoplakic patches (red or red-and-white) with slight surface irregularities and well-defined margins. On palpation, induration (firmness) may be noted, although pain is often absent or minimal [40].

In advanced stages, OSCC presents with classic features such as ulceration, nodularity, and fixation to deeper tissues. Ulcerative lesions are among the most common presentations, usually with irregular margins, raised edges, and a hardened base on palpation. Larger tumors are frequently painful, with pain sometimes radiating to the ipsilateral ear [40]. Other manifestations include exophytic growth with verrucous (warty) surfaces, poorly defined margins, and firm consistency. Less common presentations may include numbness or paraesthesia of the chin, non-healing extraction sites, or abnormal vascularized masses. Additional systemic signs can consist of dysphagia or unexplained weight loss. Advanced OSCC is frequently associated with cervical lymph node metastases; nodes that are enlarged, firm, or fixed strongly suggest metastatic spread. In approximately 5% of cases, a metastatic cervical lymph node may be the first clinical finding in the absence of an obvious primary lesion. When this occurs, the base of the tongue, tonsils, or nasopharynx are the most likely primary sites [40].

**Fig. 3:**
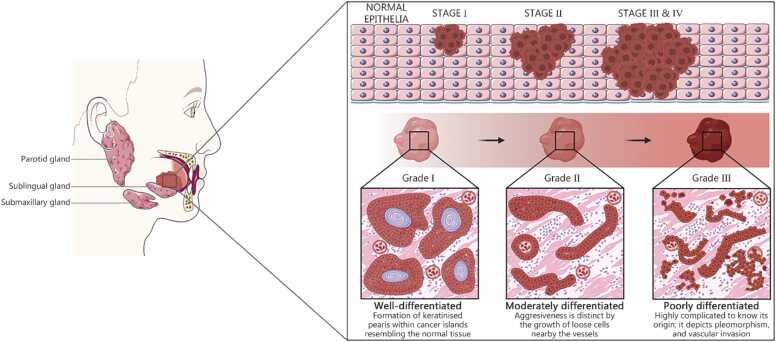
Histopathological staging and grading of OSCC progression. Histopathological presentation of OSCC, including staging and grading of OSCC solid tumors. TNM staging has been widely used for histopathological analysis of biopsy samples, where T represents the tumor size and extent of invasion around the tissues, N represents invasion of the growing tumor to the lymph node, and M represents metastasis to other organs. However, grading is different from tumor staging and is often described as I, II, or III, which represent the degree of differentiation of tumor cells from normal cells. However, TNM staging is different in laryngeal and tongue carcinomas because of differences in anatomical sites. TNM. Tumour, node, and metastasis.

Histopathological characterization of oral cancers is based on the tumour, node, and metastasis (TNM) staging and grading system, as outlined in the 8th edition of the AJCC Manual [41]. Solid oral tumors are staged by tumor size, lymph node involvement, and the presence of metastasis, and they are graded according to cellular differentiation, aggressiveness, morphology, and biological behavior [40], as illustrated in Fig. 3.

## 4 Epigenetic landscape and molecular pathogenesis of OSCC

OSCC progression is a gradual and multifaceted process shaped by intricate interactions among environmental exposures, lifestyle habits, genetic predispositions, and epigenetic alterations. Although genetic mutations remain central to neoplasm initiation, increasing evidence points to the significant contribution of epigenetic alterations that control the expression profile without mutating the fundamental DNA sequence in the pathogenesis of OSCC [42].

**Fig. 4:**
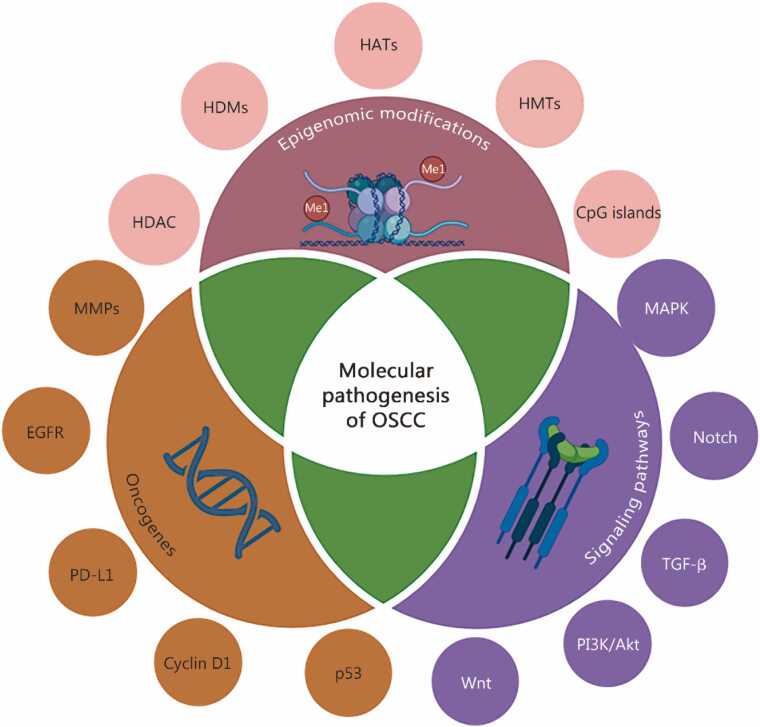
Key molecular drivers and pathways underlying OSCC development. Interplay of different factors responsible for the molecular pathogenesis of OSCC. OSCC is often associated with epigenomic alterations, which include modifications in DNA methylation, histones, signal transducers, and oncogenes, leading to the overexpression and dysregulation of the signaling cascade. HDAs. Histone deacetylases; HDMs. Histone demethylases; HATs. Histone acetyltransferases; HMTs. Histone methyltransferases; CpG. Cytosine-phosphodiester bond-guanine; MAPK. Mitogen-activated protein kinase; TGF-β. Transforming growth factor-β; PI3K/Akt. Phosphoinositide 3-kinase (PI3K)/protein kinase B; Wnt. Wingless-related integration site; P53. Tumor protein p53; PD-L1. Programmed death-ligand 1; EGFR. Epidermal growth factor receptor; MMPs. Matrix metalloproteinases.

Several established risk factors have been linked with OSCC occurrence and the advancement of the associated cancer. The etiological factors (such as tobacco chewing and alcohol abuse; tobacco smoking and betel quid chewing) often act synergistically, contributing to chronic inflammation and oxidative stress, which are implicated in DNA damage and normal cellular pathway disruption [24]. Other contributors, in addition to lifestyle habits such as tobacco and alcohol use, are genetic predispositions, poor oral hygiene, and nutritional imbalances [31]. Most importantly, not all carcinogenic exposures lead to direct genetic mutations. Many exert their influence by inducing epigenomic changes that can suppress tumor suppressor genes (TSGs) or activate oncogenes, thus increasing malignant transformation [42]. OSCC development is driven by alterations in the epigenetic landscape and two prominent molecular pathogeneses, involving oncogene activation and its aberrant signaling cascades [36]. These molecular events are interconnected and often synergistically participate in the transition from normal to neoplastic behavior, highlighting the complex biological nature of OSCC, as shown in Fig. 4**.**

### 4.1 Abrupt expression of oncogenes

OSCC is often characterized by the aberrant expression of numerous genes, either as upregulated oncogenes or downregulated tumor suppressor genes. These molecular alterations are critically involved in key cellular processes, including aberrant proliferation, resistance to cell death, tissue invasion, angiogenesis, immune system evasion, and metastatic spread. A number of these genes are consistently dysregulated in OSCC and are listed in Table 1 [43], [44], [45], [46], [47], [48], [49], [50], [51], [52], [53], [54], [55].

**Table 1:** Altered gene expression in OSCC.

Gene	Function	Role in OSCC	References
*EGFR*	Cellular growth and resistance to cell death	Frequently upregulated; Correlated with poor prognostic and therapeutic resistance	[43]
*CCND1* (Cyclin D1)	Regulation of cell cycle (G1 to S phase transition)	Upregulated; Promotes abrupt cell proliferation	[44]
*MMP-2/-9*	Invasion *via* ECM degradation	Upregulated; Promote invasion and metastasis by breaking down the basement membrane	[45]
*Bcl-2*	Anti-apoptotic protein	Upregulation leads to resistance to cell death	[46]
*TP63*	Epithelial integrity and stemness	Upregulated in OSCC, involved in cell survival and stemness	[47]
*MYC*	Transcription factor	Enhances proliferation, metabolism, and tumorigenesis	[48]
*PD-L1 (CD274)*	Immune checkpoint protein	Upregulation allows evasion of the immune system surveillance	[49]
*IL-6*	Cytokine signaling	Upregulated; Promotes immune suppression and inflammation-associated tumor growth	[50]
*TP53*	Genomic stability, apoptosis induction	Mutated or deleted in 50% – 70% of OSCCs	[51]
*CDKN2A (p16/INK4α)*	Cell cycle inhibition (*via* RB pathway)	Frequently inactivated *via* mutation or promoter methylation	[52]
*PTEN*	PI3K/Akt pathway attenuation	Loss leads to increased survival and cellular growth	[53]
*E-cadherin (CDH1)*	Cell adhesion molecule	Attenuation induces EMT and metastasis	[54]
*SOCS1*	Negative regulator of JAK/STAT	Attenuated by miR-155; Promotes immune evasion	[55]

EGFR. Epidermal growth factor receptor; MMPs. Matrix metalloproteinases; ECM. Extracellular matrix; Bcl2. B-cell lymphoma 2; TP63. Tumor protein p63; MYC. Myelocytomatosis oncogene; PD-L1. Programmed death-ligand 1; IL-6. Interleukin 6; TP53. Tumor protein 53; p16/CDKN2A. Cyclin-dependent kinase inhibitor 2 A; INK4α. Inhibitor of kinase 4α; RB. Retinoblastoma; PTEN. Phosphatase and tensin homolog; PI3K. Phosphatidylinositol 3-kinase; AKT. Protein kinase B; CDH1. Cadherin 1; EMT. Epithelial-mesenchymal transition; SOCS1. Suppressor of cytokine signaling 1; JAK. Janus kinase; STAT. Signal transducer and activator of transcription

### 4.2 Epigenomic modifications

Epigenomic modifications are dominant in both the onset and progression of OSCC. These alterations do not alter the DNA sequence but can be inherited through cell division, influencing gene expression patterns that influence the complexity, aggressiveness, and therapeutic resistance of the tumor [42]. In OSCC, three primary epigenetic mechanisms have been identified as key drivers: aberrant DNA hypermethylation, histone modifications, and post-transcriptional regulation mediated by microRNAs.

#### 4.2.1 DNA hypermethylation

Among the various epigenomic mechanisms studied in OSCC, DNA hypermethylation has received considerable attention because of its pivotal role in disease progression [42]. The process of DNA hypermethylation typically involves the accumulation of methyl groups to cytosine bases within CpG di-nucleotide islands, leading to structural alterations in chromatin that affect gene transcription. The enzymes catalyzing this modification are DNA methyltransferases (DNMTs), which utilize S-adenosylmethionine (SAM) as a methyl donor to methylate the 5th position of cytosine residues [42], [56].

When CpG-rich promoter regions undergo methylation, the expression of associated genes, particularly TSGs, is often silenced. Aberrant promoter hypermethylation of these TSGs is a regular molecular outcome in OSCC. A notable example is the *RASSF1A* gene, which encodes a protein essential for regulating the cell cycle and initiating apoptosis [42], [57]. Epigenetic silencing through gene promoter hypermethylation is recurrently detected in OSCC and contributes significantly to unchecked cellular proliferation and neoplasm growth [42], [57]. Similarly, the *APC* gene, an essential component of the Wnt signal transduction pathway, is often transcriptionally silenced due to promoter hypermethylation. This epigenetic disruption leads to the buildup of β-catenin and aberrant instigation of downstream oncogenic signals, fostering malignant transformation [57], [58]. Another gene, *MLH1*, which plays a central role in DNA mismatch repair, is also commonly affected by hypermethylation in OSCC. It’s silencing compromises DNA repair fidelity, resulting in increased genomic instability and accumulation of mutations [59]. The most frequently methylated gene in OSCC is *p16*^INK4a^ (also known as *CDKN2A*), which is involved in cell cycle regulation. Ukey *et al.* [60] promoter hypermethylation of this gene leads to cell cycle dysregulation, a finding corroborated. Environmental carcinogens, such as polycyclic aromatic hydrocarbons and nitrosamines, have been implicated in inducing epigenetic alterations [42]. The silencing of key regulatory genes such as *p16*^INK4a^ and *RASSF1A* is common in premalignant oral lesions, particularly among individuals with a history of tobacco use [61]. Hypermethylation of *p16*^INK4a^ has been reported in 40%–60% of these precancerous lesions, supporting the usefulness of *p16*^INK4a^ as an OSCC biological marker for early diagnosis [42], [62]. In addition, inhibition of the DNA repair gene *MGMT* prevents the cell from repairing from genotoxic stress, hence increasing genomic instability. The cumulative effect of these methylation-driven alterations leads to the functional suppression of tumor suppressors and other key regulators involved in apoptotic cell death, DNA damage repair, and cell cycle regulation, eventually facilitating tumor progression [42], [63].

Recent studies have shown how DNA methylation is associated with other genetic changes that can lead to cancer. This understanding paves the way for new treatments for OSCC [42], [43], [55]. Some drugs that remove methyl groups are useful in turning back on TSGs, which could lead to more focused and effective treatment options.

#### 4.2.2 Histone modifications

Histones are essential structural polypeptides that help organize and compact DNA within the nucleus by forming nucleosomes, which serve as the basic units of chromatin. Histones do more than just hold the structure together [42]. They can also be altered in different ways upon their formation, and these alterations are key signals that determine how tightly DNA is packed. This affects how easy it is for the DNA to be read to make proteins. These alterations play essential roles in controlling genes and how DNA is organized. In OSCC, such histone modifications are significantly involved in altering cellular processes vital for maintaining normal function and preventing neoplasm transformation [64].

The main types of histone alterations include acetylation, methylation, phosphorylation, ubiquitination, and sumoylation, each of which typically targets specific amino acid residues on the histone N-terminal tails [64], [65]. These chemical changes are controlled by distinct groups of enzymes: histone acetyltransferases (HATs) and histone deacetylases (HDACs) [66] govern acetylation, whereas HMTs and HDMs regulate methylation [65]. Collectively, these enzymes are central to maintaining a balance between transcriptional activation and repression [64], [65]. In OSCC, the dysregulation of these histone-modifying enzymes disrupts normal gene expression and regulation patterns, thereby promoting tumor initiation and advancement [64].

***Histone acetylation*** Histone acetylation has been explored most extensively, particularly because of its significant role in gene regulation in tumorigenesis [64]. It is catalyzed by HATs and involves the incorporation of −COCH₃ groups into Lys residues on the tails of histones. This addition tends to result in an exposed structure of chromatin and thereby promotes gene transcription. In contrast, HDACs curtail −COCH₃ groups, resulting in nucleosomal condensation and an attenuated expression profile [66]. Disruption of the stability between acetylation and deacetylation has been closely linked to the oncogenic transformation observed in OSCC. In particular, abnormal expression of HDACs has been consistently reported in OSCC and is correlated with tumor development and progression [65], [66]. The overexpression or increased activity of HDACs often leads to the epigenetic suppression of TSGs, thereby promoting uncontrolled cell growth and enabling resistance to apoptotic cell death [64]. For example, HDAC 1/2/3 are often reported to be upregulated and associated with poor differentiation and metastasis of OSCC tumors, whereas HDAC 6 dysregulates cytoskeletal dynamics, cell motility, invasion, and epithelial-mesenchymal transition (EMT) in OSCC [66]. Another silent information regulator, sirtuin 1 (SIRT1), contributes to resistance development in the OSCC tumor microenvironment (TME) [67].

Contemporary studies have emphasized the potential of HDAC inhibitors (HDACi) as therapeutic agents in OSCC [64], [66], [67]. These inhibitors are capable of reversing aberrant epigenetic changes by reactivating silenced tumor suppressor genes and triggering apoptotic responses in cancer cells. As a result, HDACi is being actively investigated as a promising epigenetic-based treatment for OSCC and other types of cancer [42].

***Histone methylation*** Histone methylation denotes a crucial epigenetic mechanism that can either promote or suppress the genetic expression profile, relying on the specific amino acid residue being altered, most commonly lysine or arginine, and the degree of methylation, whether mono-, di-, or tri-methylation [65]. In OSCC, disruptions in normal histone methylation patterns have been correlated with aberrant expression of genes integral to neoplasm development and advancement [64].

A prominent example of a suppressive histone mark is the tri-methylation of Lys 27 residues on histone H3 (H3K27me3). This alteration is catalyzed by Enhancer of Zeste Homolog 2 (EZH2), a key histone methyl-transferase (HMT) and central constituent of the Polycomb Repressive Complex 2 (PRC2) [64], [65]. In OSCC, EZH2 is often overexpressed, leading to elevated H3K27me3 levels, which are closely linked with neoplasm advancement and poor clinical outcomes. EZH2 contributes to oncogenesis by silencing TSGs, thereby promoting uncontrolled cell growth and reducing susceptibility to apoptosis [68]. Similarly, Zheng *et al.* [69] reported that high EZH2 expression suppresses critical tumor-suppressive pathways in OSCC. Conversely, inhibition of EZH2 activity has been shown to restore the function of silenced tumor suppressors and impede neoplasm growth, highlighting its potential as a theragnostic target. Suppressor of variegation 3–9 homolog 1 (SUV39H1) is reported to upregulate H3K9me3, contributing to heterochromatin formation and tumor progression, and similarly, SET domain containing 2 (SETD2) also alters the same site to promote genetic instability [64]. Another HMT, i.e., G9a or EHMT2, promotes OSCC pathogenesis by silencing TSGs through association with H3K9me2 [64], [69]. Moreover, the use of histone methyl-transferase inhibitor (HMTi), which is able to reverse malignant phenotypes and reactivate epigenetically silenced tumor suppressor genes, has yielded encouraging outcomes in preclinical studies of OSCC [69].

Given these promising findings, further investigations are warranted, particularly in conjunction with traditional therapeutics such as chemotherapeutics or immunotherapies, to assess their therapeutic value fully and improve the clinical outcomes of OSCC patients [42].

#### 4.2.3 Post-transcriptional regulation by miRNAs

miRNAs are short, non-coding, single-stranded RNA sequences, typically 18–25 nucleotides in length, which play a critical role in regulating gene expression post-transcriptionally usually function by attaching to complementary nucleotide chains in the 3’ untranslated regions (*3’UTRs*) of target mRNAs, leading either to the suppression of protein synthesis or degradation of the mRNA, depending on how well the sequences match. These molecules have emerged as central regulators in an extensive range of biological and pathological processes, including those critical to OSCC, such as cell multiplication, programmed cell death, angiogenesis, and metastasis [70], [71].

Research has revealed numerous miRNAs with altered expression in OSCC. Among them, miR-21 is consistently reported to be upregulated and acts as an oncogenic miRNA or an oncomiR. It promotes tumor cell survival and blocks apoptosis by downregulating key tumor suppressors, such as phosphatase and tensin homolog (PTEN) and programmed cell death protein 4 (PDCD4). Elevated miR-21 levels have been linked with poor clinical outcomes in patients with OSCC [71]. In addition, miR-21 influences the TME by directing tumor-associated macrophages (TAMs) toward a pro-tumorigenic phenotype, facilitating immune system evasion, and supporting tumor progression. miR-21 also enhances the resistance of cancer cells to oxidative stress, thereby helping them withstand treatments such as radiotherapy [70], [71].

In contrast, miR-34a attenuates neoplasms and is a direct transcriptional target of tumor protein p53 (TP53). It has a substantial effect on halting cell division, inducing apoptosis, and limiting cell proliferation. Especially in OSCC, miR-34a is frequently silenced *via* epigenomic mechanisms such as promoter gene hypermethylation, leading to its reduced expression and contributing to tumor progression and poor patient prognosis. Additionally, miR-34a helps inhibit EMT by targeting EMT-associated genes such as *SNAI1* and *ZEB1* [72].

Another key miRNA, miR-155, is often amplified in OSCC and promotes tumor growth and invasion by suppressing tumor suppressors such as suppressor of cytokine signaling 1 (SOCS1) and CCAAT/enhancer binding protein β (C/EBPβ). SOCS1 negatively regulates the JAK/STAT-mediated signal transduction cascade, which is central to immunogenic responses and cancer progression. Furthermore, by downregulating C/EBPβ, a transcriptional component involved in immune regulation and cellular differentiation, miR-155 exacerbates tumor aggressiveness [73].

miR-200c also has a substantial regulatory effect on OSCC, particularly in controlling EMT. Reduced expression of miR-200c is often linked with enhanced neoplasm metastatic activity and invasiveness. This occurs through the upregulation of its target ZEB1/2, which suppresses epithelial characteristics such as E-cadherin expression and promotes mesenchymal traits such as increased motility and resistance to cell death. These changes collectively facilitate cancer cell invasion and dissemination throughout the body [74].

### 4.3 Aberrant signaling pathways in OSCC

OSCC arises from intricate dysregulation of various cellular pathways that govern fundamental bioprocesses, such as those involved in cellular division, differentiation, death, malignancy progression, and angiogenesis [75]. The dysregulation of these signaling networks is highly important for initiating tumorigenesis, driving tumorigenesis progression, and promoting resistance to treatment [75]. Among the most frequently disrupted pathways are the epidermal growth factor receptor (EGFR) mediated mitogen-activated protein kinase (MAPK) signaling pathway, which induces dysregulated cell growth [76]. Similarly, the PI3K/Akt/mTOR signaling pathway is usually overexpressed, allowing cancer cells to exist and proliferate under unfavorable circumstances [75]. Other pathways vital for normal tissue maintenance, including Wnt-mediated β-catenin and Notch signaling, are commonly disrupted in OSCC, resulting in aberrant cell differentiation and enhanced self-renewal capabilities, features often associated with cancer stem cells (CSCs) [77].

#### 4.3.1 MAPK signaling in OSCC

The MAPK signaling cascade is pivotal for the regulation of several essential cellular activities, including multiplication, differentiation, survival, and apoptosis [77], [78]. Under normal physiological conditions, MAPK signaling is initiated through a downstream cascade involving MAPK kinase kinase (MAPKKK), MAPK kinase (MAPKK), and MAPK itself [78]. This cascade system is typically instigated by external signaling molecules involving cytokines, cellular stress, or mitogenic factors. Upon stimulation, MAPKKK activates MAPKK, which in turn phosphorylates MAPK. The activated MAPKs then either migrate to the nucleus or interact with targets in the cytoplasm, triggering alterations in gene expression and functional intracellular responses. The major MAPK subfamilies, such as c-Jun N-terminal kinase 1/2/3 (JNK1/2/3), extracellular signal-related kinase 1/2 (ERK1/2), and p38α/β/γ/δ, mediate context-specific responses, tailoring cellular outcomes to various physiological and pathological stimuli [78], [79], as shown in Fig. 4**.**

In OSCC, this signaling pathway becomes deregulated and significantly contributes to both cancer initiation and progression [77], [78], [79]. The ERK1/2 pathway, which is commonly linked with cell proliferation and survival, is frequently overactivated in OSCC. This leads to the upregulation of oncogenic molecules such as c-Myc, vascular endothelial growth factor (VEGF), and matrix metalloproteinases (MMPs), all of which drive uncontrolled cell division, new blood vessel formation, and degradation of the extracellular matrix (ECM), facilitating tumor expansion and metastasis [79]. However, the JNK and p38-mediated MAPK pathways, which are typically involved in the regulation of cell stress and programmed cell death induction, orchestrate dual functions in OSCC. These pathways may inhibit or promote neoplastic growth via the TME and stimuli [77]. For example, activated JNK can induce apoptosis in OSCC cells; however, in certain settings, it may also promote tumorigenesis through crosstalk with pro-survival pathways such as the signal transducer and activator of transcription 3 (STAT3) and nuclear factor kappa-light-chain-enhancer of activated B cells (NF-κB) pathways [79].

Genetic alterations in MAPK pathway components are common in OSCC. Notably, *MAPK1* mutations, which are particularly prevalent in Asian populations with HNSCC, are linked with poor clinical outcomes [78], [79]. Aberrant activation of MAPK subtypes, including p38, ERK1/2, and JNK, has also been frequently identified in tumor samples. Specifically, p38 activation is correlated with tumor aggressiveness, accompanied by limited survival [77], [79]. Moreover, MAPK signaling also influences EMT and metastasis by regulating p38 and ERK pathways, regulating epithelial-mesenchymal transition (EMT) biomarkers such as Snail and E-cadherin, in response to inflammatory cytokines and immune checkpoint molecules such as programmed death-ligand 1 (PD-L1), further enhancing OSCC invasiveness and metastatic potential [79], [80], [81].

#### 4.3.2 Wnt signaling in OSCC

The Wnt signaling cascade is essential for embryogenesis, maintaining tissue equilibrium, and orchestrating crucial cellular functions, including growth, differentiation, and the renewal of stemness [77], [82]. It functions primarily through two interconnected pathways, the canonical (β-catenin-dependent) pathway and the non-canonical pathway, which include the Wnt/Ca²⁺ and planar cell polarity (PCP) pathways. In the canonical route, Wnt ligands interact with Frizzled receptors and Lipoprotein receptor-related protein (LRP5/6) coreceptors, leading to nuclear sorting and β-catenin stability, where it modulates target gene expression [82]. When Wnt ligands are absent, β-catenin is continuously degraded by a degradation complex composed of Axin, glycogen synthase kinase-3 beta (GSK3β), Adenomatous polyposis coli (APC), and Casein kinase 1α (CK1α), thereby suppressing unwanted gene transcription [83]. The non-canonical/β-catenin-independent Wnt signaling cascade is activated by ligands such as Wnt5a and Wnt11, further following the Wnt/Ca^2+^ and PCP cascade signaling systems and influencing a range of cellular behaviors, including polarity, motility, and calcium signaling [77], [82]. Key downstream targets of this branch involve kinases and transcription factors such as JNK, protein kinase C (PKC), and Nuclear factor of activated T-cells (NFAT), which mediate diverse functional outcomes [83].

In OSCC, dysregulation of both the canonical and non-canonical Wnt signaling branches is common and is closely tied to tumor development [77], [82], [83], [84], [85]. In particular, aberrant canonical signaling causes the nuclear accumulation of β-catenin, which drives the expression of oncogenic targets such as c-Myc, cyclin D1, and MMP factors that support uncontrolled proliferation, invasive behavior, and resistance to apoptosis [84], [85]. While mutations in central pathway components such as APC or β-catenin are relatively uncommon in OSCC, β-catenin stabilization often occurs through alternative mechanisms, such as the epigenetic silencing of Wnt inhibitors [including Dickkopf-related protein 3 (DKK3), Wnt inhibitory factor 1 (WIF1), and secreted frizzled-related protein 2 (SFRP2)] and interactions with other oncogenic pathways, such as the EGFR signaling pathway, which can increase β-catenin stability through phosphorylation [83], [86], as shown in Fig. 4**.** Non-canonical Wnt signaling also contributes significantly to OSCC pathobiology. Wnt5a and Wnt5b, for example, are frequently overexpressed and have been linked to increased migratory and invasive capacities, as well as filopodium formation, especially in laryngeal SCCs [87]. In OSCC, Wnt5a-mediated activation of the Wnt/Ca²⁺/PKC pathway facilitates similar aggressive traits, promoting cellular motility and invasiveness [88]. Wnt5b expression is markedly elevated in highly metastatic OSCC cell lines, where it functions *via* the PCP and Wnt/Ca²⁺ arms to reorganize the cytoskeleton and augment cell movement [83].

Beyond its role in tumor progression, Wnt signaling also underpins the biology of CSCs in OSCC. Activation of the canonical pathway reinforces CSC self-renewal and survival, whereas non-canonical Wnt ligands contribute to therapy resistance through interactions with critical signaling cascades such as PI3K/Akt and yes-associated protein/transcriptional co-activator with PDZ-binding motif (YAP/TAZ) [89].

#### 4.3.3 PI3K/Akt/mTOR signaling in OSCC

The PI3K/Akt/mTOR signaling axis is commonly referred to as the PI3K/Akt/mTOR pathway and is an evolutionarily conserved intracellular regulatory signaling cascade that plays a pivotal role in regulating cell survival, proliferation, metabolism, and growth [77], [90]. Under normal conditions, this pathway is initiated through the activation of RTKs or GPCRs, which in turn activate PI3K. PI3K catalyzes the conversion of phosphatidylinositol 4,5-trisphosphate (PIP2) into phosphatidylinositol 3,4,5-trisphosphate (PIP3), a reaction that mobilizes Akt to the cell membrane. Akt is then activated through PDK1/mTORC2 phosphorylation. Once activated, Akt targets a variety of downstream effectors, such as GSK3, forkhead box O (FOXO), mouse double minute 2 homolog (MDM2), and tuberous sclerosis complex 2 (TSC2), which together promote cell cycle progression, suppress apoptosis, and support anabolic and metabolic functions [77]. The activity of this pathway is tightly modulated by tumor suppressors such as PTEN, which dephosphorylates PIP3, thus serving as a crucial brake to prevent overactivation and maintain cellular equilibrium [90].

The PI3K/Akt/mTOR pathway is frequently altered, contributing substantially to tumor development, progression, and resistance to treatment in OSCC and other malignancies [77]. Common molecular disruptions include gain-of-function mutations in 3-kinase catalytic subunit alpha (PIK3CA), which encodes the p110α catalytic subunit of PI3K; amplification or mutation of Akt isoforms; and inactivation of PTEN through genetic or epigenetic mechanisms [77], [90]. These aberrations result in pathway activation that is sustained, promoting uncontrolled cellular proliferation, metabolic changes that favor tumor development, and heightened resistance to cell death [77]. Notably, hypermutable regions in PIK3CA, such as E545K and H1047R, which are frequently observed in HNSCC, activate PI3K signaling through either attenuating regulatory inhibition or fostering membrane interactions [90]. Concurrently, the loss of PTEN removes a critical inhibitory checkpoint, further driving hyperactivation of the signaling cascade [77], [90]. Increased Akt signaling not only suppresses apoptosis by inhibiting proapoptotic proteins such as BAD and FOXO but also stimulates antiapoptotic regulators such as Mouse double minute 2 homolog **(**MDM2) and X-linked inhibitor of apoptosis protein (XIAP) [90]. This dysregulation propagates through mammalian target of rapamycin complex 1 (mTORC1), which governs processes such as protein synthesis, cellular metabolism, and autophagy. When overactivated, mTORC1 promotes tumor growth and processes such as EMT, invasion, and metastasis in OSCC [77], [90], as shown in Fig. 4**.**

Crucially, the PI3K/Akt/mTOR pathway interacts with other key signaling cascades, including the Ras/ERK and Wnt-mediated β-catenin pathways [77]. This extensive crosstalk amplifies oncogenic signaling and contributes to therapeutic resistance, thereby driving OSCC toward more aggressive and treatment-refractory stages [90].

#### 4.3.4 Notch signaling in OSCC

The Notch signaling cascade is an evolutionarily conserved intercellular communication that plays a fundamental role in governing crucial cellular functions, including differentiation, proliferation, death, and stemness [91]. In mammals, this pathway operates through 4 receptors (Notch1–4) and 5 ligands (Jagged 1, 2, and Delta-like 1, 3, and 4). Upon extracellular ligand engagement, the Notch receptor is subjected to a sequence of proteolytic cleavages, the final of which is transduced by γ-secretase. This cleavage releases the Notch intracellular domain (NICD), which is sorted into the nucleus. The NICD assembles a transcriptional complex with CSL and associated coactivators, ultimately driving the expression profile of downstream target genes such as *Hes1, Hey1*, and *c-Myc*. These genes orchestrate diverse biological effects, depending on the specific cell type [91], [92].

In OSCC, the Notch pathway has dual functions as either a tumor attenuator or a promoter. During the early process of carcinogenesis, Notch1 normally possesses tumor-suppressing activity through the promotion of epithelial differentiation and preservation of normal tissue structure [92]. Loss-of-function mutations in Notch1 are recurrently identified in HNSCC, including OSCC, and are associated with impaired differentiation and uncontrolled cell proliferation, which are hallmarks of malignant transformation [91]. However, in advanced OSCC, the pathway may shift toward oncogenic behavior. Upregulated levels of Notch receptors and ligands have been detected in OSCC specimens and are associated with adverse clinical outcomes, including increased angiogenesis, increased metastatic potential, and resistance to conventional therapies. Notably, overactivation of Notch1 and Notch3 has been associated with the promotion of EMT, a critical step in tumor invasion and dissemination. Furthermore, Notch signaling can synergize with other oncogenic pathways, such as the NF-κB, PI3K/Akt/mTOR, and Wnt/β-catenin pathways, thereby compounding tumorigenic signaling cascades [93].

**Fig. 5:**
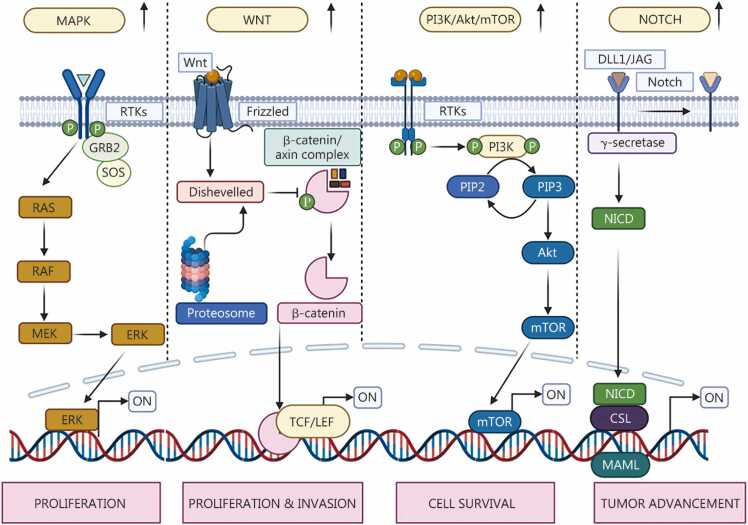
Dysregulated signaling pathways regulating proliferation, survival, advancement, and invasion in OSCC. Altered oncogenic MAPK, WNT, PI3K/Akt/mTOR, and NOTCH signaling pathways are involved in cancer metastasis. These dysregulated signaling pathways are often regulated by different dysregulated/mutated signal transducers, which further lead to evasion of cell death, promoting cell survival and allowing aberrant cell proliferation, metastasis, and tumor advancement. MAPK/MEK. Mitogen-activated protein kinase; RTKs. Receptor tyrosine kinases; GRB2. Growth factor receptor-bound protein 2; SOS. Son of sevenless; RAS. Rat sarcoma; RAF. Rapidly accelerated fibrosarcoma; ERK. Extracellular signal-regulated kinase; WNT. Wingless-related integration site; TCF. T-cell factor; LEF. Lymphoid enhancer-binding factor; PI3K. Phosphatidylinositol 3-kinase; PIP2. Phosphoinositide 2-kinase; PIP3. Phosphoinositide 3-kinase; Akt. Protein kinase B; mTOR. Mammalian target of rapamycin; DLL1. Delta-like 1; JAG. Jagged; NICD. Notch intracellular domain; CSL. Suppressor of hairless lag 1; MAML. Mastermind-like; ON. Indicates activation of the downstream target gene transcription.

Dysregulation of the Notch signaling pathway also orchestrates a key function in sustaining CSCs within OSCC. These CSCs are distinguished by their self-renewal capacity, contribution to tumor relapse, and innate resistance to both chemotherapy and radiotherapy [77], [93]. Activation of Notch signaling, particularly *via* Notch1 and Jagged1, has been shown to support CSC maintenance, tumor advancement, and survival, underscoring the ability of this pathway as a target for OSCC therapeutic intervention [91], [92], as shown in Fig. 5**.**

#### 4.3.5 Transforming growth factor-β signaling in OSCC

TGF-β is a multifunctional cytokine involved in the modulation and regulation of numerous cellular processes, including division, differentiation, apoptosis, and immune modulation [94], [95], [96]. Under physiological conditions, TGF-β controls tissue homeostasis and acts as a tumor suppressor, particularly in epithelial tissues. Canonical TGF-β signaling begins with ligand binding to TGF-β receptor type II (TβRII), which recruits and phosphorylates TGF-β receptor type I (TβRI). This receptor complex activates suppressor of mothers against decapentaplegic homolog (SMAD)2 and SMAD3, which then couple with SMAD4 and translocate to the nucleus to regulate target gene expression. In addition to this SMAD-dependent pathway, TGF-β can activate non-canonical (SMAD-independent) pathways such as the MAPK, PI3K/Akt/mTOR, and Rho-like GTPase signaling pathways, contributing to context-dependent cellular outcomes [94].

In OSCC, TGF-β signaling has dual functions, exerting an anti-tumor effect in early stages and promoting tumor development in advanced disease. During early carcinogenesis, TGF-βs suppress epithelial cell cycle progression by inducing G1 arrest, suppressing oncogenes such as *MYC*, and upregulating CDKIs such as *p15* and *p21*. These mechanisms collectively contribute to growth arrest and apoptosis in early-stage tumor cells [95], [96], [97]. However, as tumorigenesis progresses, OSCC cells often acquire resistance to TGF-β-induced growth inhibition due to mutations in or downregulation of key signaling components, such as TβRII, SMAD2, or SMAD4. Loss of TβRII expression has been correlated with increased invasiveness and poor prognosis in OSCC patients [94], [98], [99] by downregulating epithelial markers and upregulating mesenchymal markers through transcription factors such as Snail, Slug, and Twist [96]. In addition, TGF-β causes the activation of matrix metalloproteinases (e.g., MMP-9), which can disrupt ECM components, thereby augmenting the spread of the tumor [94].

TGF-β also impairs immune surveillance in OSCC by suppressing cytotoxic T-cell responses and NK cell activity, in part through the downregulation of IL-2 and NK cell receptors. It induces an immunosuppressive TME by promoting regulatory T-cell differentiation and M2 macrophage polarization, thereby facilitating tumor progression and resistance to therapy [94].

Hence, understanding these aberrant signaling mechanisms is crucial for pinpointing potential therapeutic targets and prognostic and diagnostic biomarkers, and developing precision medicine approaches. Targeted therapies aimed at correcting or inhibiting specific signaling abnormalities hold promise for improving outcomes in oral cavity cancer, particularly when combined with traditional modalities such as surgery, radiation, and chemotherapy [75].

## 5 OSCC hallmarks and associated biomarkers

The term “hallmarks of cancer” refers to the biological characteristics of cancer, which were introduced by Douglas Hanahan and Robert Weinberg and are immensely helpful in both understanding cancer biology and guiding research and therapy development. The hallmark framework divides this complexity into distinct biological capabilities that serve as a foundational paradigm in oncology and a strategic blueprint for innovation in cancer research, diagnosis, and treatment, as shown in Table 2 [100], [101], [102], [103], [104], [105], [106], [107], [108], [109], [110], [111], [112], [113], [114], [115], [116], [117], [118], [119], [120], [121], [122], [123], [124], [125], [126], [127], [128], [129], [130], [131], [132], [133], [134], [135].

**Table 2:** OSCC hallmarks and their associated biomarkers.

**Hallmarks of OSCC**	**Associated biomarkers**	**References**
Sustaining proliferative signaling	EGFR (HER1-4), STATs, Cyclin D1, c-Met, p7056k, Akt, mTOR	[100], [101], [102]
Evading growth suppressors	p53, p27, p16, p21, PTEN	[103], [104], [105]
Resisting cell death	Fas, FasLG, FADD, Bcl2, Bax, Survivin, cIAP2, TRAIL	[106], [107], [108], [109], [110], [111], [112]
Enabling replicative immortality	hTERT	[113]
Activating invasion and metastasis	E-cadherin, N-cadherin, β-catenin, Twist, Zeb 1/2, SNAIL, Slug, MMP-7/11/13/21, Vimentin	[112], [113], [114], [115], [116], [117], [118]
Inducing angiogenesis	VEGF A/C/D	[119]
Genome instability and mutation	LOH and genome-wide alterations in 3p, 9p (p16 inactivation), 17p (p53 inactivation), 4q, 8p, 11q, 13q, H-Ras	[120], [121]
Tumor-promoting inflammation	MMPs, lymphocytes count, COX-2, IL-6/8, TNF-α, CD44, CD68^+^, CD163^+^	[122], [123], [124], [125], [126]
Avoiding immune destruction	PD-1, PD-L1	[127]
Deregulating cellular energetics	HIF1α, GLUT1	[128], [129], [130]
Unlocking phenotypic plasticity	CD24^+^	[131]
Non-mutational epigenetic reprogramming	*p16*^INK4a^, *APC, MGMT, CDH1* are aberrantly methylated TSGs	[132]
Polymorphic microbiomes	*Fusobacterium periodonticum*, *Parvimonas micra*, *Streptococcus constellatus*, *Haemophilus influenzae*	[133], [134]
Senescent cells	p21, p16, pRB, Maspin, G-actin, p15	[135]

EGFR. Epidermal growth factor receptor; HER. Human epidermal growth factor receptor; STAT. Signal transducer and activator of transcription; c-Met. Cellular mesenchymal epithelial transition; p70S6K. p70 ribosomal protein S6 kinase; Akt. Protein kinase B; mTOR. Mammalian target of rapamycin; p53. Tumor protein 53; PTEN. Phosphatase and tensin homolog; Fas. Fas cell surface death receptor; FasLG. Fas ligand; FADD. Fas-associated death domain; Bcl2. B-cell lymphoma 2; BAX. Bcl2-associated X protein; cIAP2. Cellular inhibitor of apoptosis protein 2; TRAIL. Tumor necrosis factor-related apoptosis-inducing ligand; hTERT. Human telomerase reverse transcriptase; ZEB1/2. Zinc finger E-box binding homeobox; SNAIL. Snail family transcriptional repressor 1; MMP. Matrix metalloproteinase; VEGF. Vascular endothelial growth factor; LOH. Loss of heterozygosity; H-Ras. Harvey rat sarcoma viral oncogene homolog; COX2. Cyclooxygenase-2; IL. Interleukin; TNF-α. Tumor necrosis factor-α; CD44. Cluster of differentiation 44; PD-1. Programmed cell death protein 1; PD-L1. Programmed death-ligand 1; APC. Adenomatous polyposis coli; MGMT. O^6^-methylguanine-DNA methyltransferase; CDH1. Cadherin 1; TSGs. Tumor suppressor genes; HIF1α. Hypoxia-inducible factor 1 alpha; GLUT1. Glucose transporter 1

## 6 Interplay of cancer-associated fibroblasts in immunomodulation in the TME

The TME is a dynamic, complex microenvironment that contains cancer cells, stromal cells, immunogenic cells, blood vessels, the ECM, and numerous signaling molecules [6]. One of the distinguishing features of the OSCC microenvironment is the extensive remodeling of the ECM, which facilitates cancer cell invasion and metastasis [6]. Immunoprofiling in OSCC has provided significant insights into the dynamic interaction between cancer cells and the immune microenvironment, highlighting that cancer-associated fibroblasts (CAFs) are critical contributors to tumor progression [136]. CAFs have the capacity to modify the immune landscape in OSCC by influencing the recruitment of dense ECM, hindering functional characteristics of T lymphocytes, macrophages, and natural killer cells by secreting cytokines and immunosuppressive chemokines [6]. Thereby establishing an immunosuppressive environment conducive to tumor growth and metastasis in the TME through tumor-associated macrophages (TAMs), myeloid-derived suppressor cells (MDSCs), and polarized macrophages (M2 pro-tumorous phenotype) [6], [136].

Notably, CAFs produce TGF-β and IL-6, contributing to a macrophage phenotype that favors tumor advancement and diminishes cytotoxic T-cell responses, thereby shifting the dynamic from immune surveillance to immune evasion [137]. The immunosuppressive effects of CAFs also extend to their regulation of immune checkpoint pathways, a key mechanism by which tumors evade immune destruction [137]. In the context of OSCC, CAFs can increase the expression of checkpoint molecules, including PD-L1 and cytotoxic T-lymphocyte-associated protein 4 (CTLA-4) ligands, which either directly or indirectly exacerbate T-cell exhaustion within the TME [136]. This observation is particularly significant given that immune checkpoint inhibitors targeting PD-1/PD-L1 and CTLA-4 have become fundamental treatments for various cancers. However, their effectiveness in OSCC has been inconsistent, which is partially due to the antagonistic influence of CAFs [137]. The ability of CAFs to modulate TGF-β/SMAD, Stromal cell-derived factor 1 (SDF-1)/C-X-C chemokine ligand 12 and C-X-C chemokine receptor type 4 (CXCL12-CXCR4) axis [137], IL-6/JAK/STAT3, PD-1/PD-L1 [136], and Wnt signaling pathways suggests that their presence may indicate resistance to checkpoint blockade, thereby underscoring the importance of integrating stromal-targeted approaches with immunotherapies [6], [137]. Immunoprofiling in this context revealed that OSCCs enriched in CAFs frequently exhibit elevated levels of exhausted T cells and immunosuppressive macrophages, indicating the direct impact of stromal remodeling on immune evasion [136], [137].

The anti-immune effects of CAFs in OSCC are, therefore, intricate and involve both biochemical and structural mechanisms that diminish the efficacy of host immunity [136]. Through the secretion of soluble factors, CAFs impede dendritic cell maturation, disrupt antigen presentation, and promote the expansion of regulatory T cells, thereby systematically weakening adaptive immunity [137]. Additionally, the dense ECM produced by CAFs restricts immune cell movement, further reducing the likelihood of effective tumor immune surveillance [6]. Collectively, these actions not only facilitate the progression of OSCC but also limit the success of immunotherapeutic approaches. Hence, although immune checkpoint inhibitors represent substantial advancements in the treatment of OSCC, their success is contingent upon overcoming the resistance posed by CAFs [6], [136].

Angiogenesis, or the formation of new blood vessels, is also a critical component of the OSCC TME. VEGF and other proangiogenic factors are overexpressed to support tumor growth and nutrition [136]. Hypoxia, a byproduct of rapid tumor growth and inefficient vascularization, also remodels gene expression and promotes aggressive tumor behavior [6].

Thus, the OSCC TME has become a crucial factor in early disease screening, biomarker detection, and timely therapeutic administration [6]. Additionally, current research emphasizes the need for combinatorial therapeutic strategies that integrate immunotherapies with agents designed to promote stromal reprogramming, which could exploit the vulnerabilities and lead to enhanced and more durable antitumor responses in patients with CAF-rich OSCC tumors [136], [137]. Clinical trials utilizing PD-1 inhibitors such as nivolumab and pembrolizumab have produced encouraging results in select cohorts of OSCC patients; however, overall response rates continue to be limited. This has prompted increased emphasis on the tumor stroma, particularly CAFs, whose secretory products, including IL-6, stromal-derived factor-1 (SDF-1), and VEGF, concurrently inhibit cytotoxic lymphocyte function and promote angiogenesis and fibrosis, thus obstructing immune cell infiltration [136]. Similarly, various other targets are also being identified to mitigate immunosuppression in the TME. Research by Heide *et al.* [138] revealed that nicotinamide N-methyltransferase (NNMT) is a central CAF regulator and represents a promising therapeutic target for TME mitigation. Hypomethylation of H3K27me3 induced by NNMT results in the secretion of complement from TME-resident CAFs, which attract immunosuppressive MDSCs to tumors. In immunocompetent mice, *NNMT* knockout hinders tumor growth in syngeneic models of ovarian, breast, and colon cancer by promoting enhanced activation of CD8^+^ T cells.

Consequently, combination strategies are being developed to simultaneously inhibit checkpoint pathways while neutralizing CAF-driven immunosuppressive signals. For example, targeting TGF-β signaling has been proposed as a strategy to reprogram the stromal compartment and enhance the efficacy of immune checkpoint therapies, whereas inhibiting IL-6 or CXCL12 signaling may dismantle barriers created by CAFs that impede T-cell infiltration [137].

## 7 Metabolic reprogramming and therapeutic resistance in OSCC

Therapeutic resistance has increasingly been recognized as a critical hallmark of the progression of OSCC and a significant factor contributing to therapeutic resistance [139]. In conjunction with immune components such as Tregs and stromal elements, tumor cells continuously modify their energy metabolism to survive in the hypoxic and nutrient-deficient TME [140]. A prominent feature of this metabolic adaptation is the preferential reliance on aerobic glycolysis, often referred to as the Warburg effect. This phenomenon facilitates rapid ATP production and the generation of biosynthetic precursors while also resulting in lactate accumulation, which enhances the immunosuppressive functions of Tregs and myeloid-derived suppressor cells. This metabolic transformation is intricately associated with resistance to various pharmacological agents [140], [141].

Cisplatin, the standard chemotherapeutic agent utilized in the treatment of OSCC, exemplifies the therapeutic resistance that arises from metabolic modifications. OSCC cells exhibiting cisplatin resistance display increased glycolytic flux, increased utilization of glutamine, and altered mitochondrial dynamics, all of which work together to maintain redox balance and bolster DNA repair mechanisms, thereby reducing the cytotoxic effects associated with cisplatin [140], [141]. Similarly, resistance to 5-fluorouracil has been linked to increased serine and one-carbon metabolism, which facilitates sustained nucleotide synthesis, even under conditions of drug exposure [142], [143]. Furthermore, resistance to methotrexate has been correlated with the upregulation of folate and purine metabolic pathways, which support both DNA synthesis and immune evasion strategies [142].

Targeted therapies and metabolic inhibitors have also faced considerable challenges related to resistance. The initial promise of employing 2-deoxy-D-glucose (2-DG) to inhibit glycolysis in head and neck cancers has been tempered by the compensatory activation of fatty acid oxidation (FAO) and the upregulation of the PI3K/Akt/mTOR signaling pathway. These adaptations enable Tregs and tumor cells to sustain their energy supply, ultimately undermining therapeutic efficacy [141]. Additionally, resistance to immune checkpoint inhibitors, such as those targeting PD-1, has been associated with the metabolic plasticity of Tregs, where fatty acid uptake through CD36 and activation of the kynurenine pathway allow for the maintenance of immunosuppressive activity despite therapeutic blockade [140], [142]. Moreover, IDO inhibitors, including epacadostat, have demonstrated limited efficacy in certain clinical trials because of the presence of redundant amino acid metabolic circuits that circumvent tryptophan restriction [140], [141], [142].

Recent research has indicated that resistance to poly (ADP-ribose) polymerase (PARP) inhibitors, such as olaparib, in OSCC may arise from increased oxidative stress tolerance and increased nucleotide synthesis within the TME. Even statins, including atorvastatin, and modulators of FAO, such as etomoxir, encounter resistance due to the heterogeneity of tumor cells and their adaptive metabolic switching abilities [140], [141], [142], [143].

The therapeutic resistance observed in OSCC exemplifies the remarkable metabolic adaptability of both tumor and immune cells, which utilize glycolysis, FAO, amino acid metabolism, and nucleotide biosynthesis in overlapping and compensatory manners. This inherent complexity elucidates why monotherapies targeting isolated pathways often yield suboptimal outcomes and suggests that combinatorial approaches targeting multiple metabolic pathways, alongside traditional therapeutic agents, may be essential for overcoming resistance in the context of OSCC.

## 8 Early OSCC screening and current diagnostic aids

OSCC is diagnosed through various invasive and non-invasive diagnostic aids, which help to diagnose OSCC in the early stages, to reduce morbidity and mortality. Timely diagnosis aids in increasing the survival of OSCC patients [144].

An OSCC screening drive with a randomized controlled trial was conducted in Kerala, India, between 1994 and 2009, which aimed to improve the OSCC stage at diagnosis and reduce mortality in the examined population. This proactive initiative covered over 96,517 participants, with a control group of 95,356 participants having no prior history of OSCC [145], [146]. During this period, a quad round of screening was performed in which a major 91% of the target population size was screened once, and after the third round of screening, a substantial reduction in mortality of over 30% was detected in high-risk OSCC patients. Furthermore, there has been an over 80% mortality reduction and an almost 40% reduction in the incidence of OSCC in the population screened compared with the control group [145], [146].

Another 5-year OSCC screening initiative was conducted between 2004 and 2009, with more than 2 million adult smokers/betel consumers, among whom 51% underwent thorough dental checkups [147]. A total of 4110 subjects among this large population were confirmed to have OSCC (stage I 46.5% and stage II, 39.6%) at the first screening test. A further 26% reduction in mortality and incidence was reported in the screened group [147]. These screening programs suggest the need for OSCC screening to reduce the burden of OSCC on healthcare and improve health. Therefore, various diagnostic methods are being used to identify precancerous and cancerous lesions.

Recent OC mass screening initiatives employed risk-stratified and technology-assisted approaches to enhance detection efficacy and feasibility. A community screening program conducted in rural regions of Varanasi district, India (2021-2023) to screen 10,101 high-risk population through smartphone-supported intraoral visual examination and imaging. Over 55% individuals were reported to have tobacco abuse. Histopathological validation of OC and OPMD was achieved on 21% individuals (i.e., >50 individuals) with suspected lesions [148].

Another cross-sectional population-based OC screening initiative was conducted in Kamrup District of Assam, Northeast India, between 2018 and 2022, with over 14,749 individuals (age≥35 years) with oral visual inspection, autofluorescence, and questionnaires by trained healthcare providers and dentists [149]. Over 1384 oral lesions were identified by dentists, in which 268 were benign lesions, 795 were tobacco pouch keratosis, and 321 OPMD cases with 8 invasive OC incidences were screened. Internally validated risk prediction model demonstrated good discrimination (AUC=0.83) with the highest risk prediction of over 30% participants accounting 81.8% of detected lesions, though external validation showed reduced performance [149].

### 8.1 Non-invasive oral cancer screening-techniques

#### 8.1.1 Conventional oral examination

Traditional methods for oral cancer screening are based primarily on visual inspection of the buccal cavity with the aid of normal incandescent lighting. While this method has long been the basis for oral evaluation, its efficiency in detecting oral cancer remains controversial. Although clinical examination can aid in identifying suspicious lesions, it often fails to differentiate between benign, harmless-appearing abnormalities and lesions with malignant potential. Consequently, premalignant changes may be overlooked, leading to missed opportunities for early intervention [150].

#### 8.1.2 Vital tissue staining

Vital staining techniques have long been utilized to aid in the prompt detection of malignant OSCC lesions, with toluidine blue staining specifically applied to oral lesions for more than three decades [150], [151]. Toluidine blue, an acidophilic dye belonging to the thiazine group, selectively stains acidic cellular components such as nucleic acids [152]. In dysplastic and cancerous oral lesions, the uptake of the dye is increased due to several pathological changes, including increased mitotic activity, elevated nucleic acid levels, disruption of cellular cohesion, and the formation of wider intercellular spaces, all of which facilitate deeper dye penetration. A lesion exhibiting dark blue coloration (either partially or entirely) is considered positive for potential malignancy [151]. Toluidine blue has demonstrated high sensitivity in detecting oral cancer, ranging between 93.5% and 97.8%, along with a specificity of 73.3% to 92.9% [152]. While the dye performs reliably in identifying carcinomas, it detects only approximately 50% of dysplastic changes or benign conditions [153].

#### 8.1.3 Exfoliative cytology

From 1955 to 1975, numerous studies explored exfoliative cytology; its use is often debated and has declined afterward because of a high false negative rate [150]. Exfoliative cytology works on the principle that when a neoplasm is present, the underlying epithelial cells lose their cohesion. This allows deeper cells, along with superficial ones, to be scraped and examined microscopically. Its benefits include being a non-invasive, quick, and straightforward procedure, making it comfortable for patients with other systemic illnesses [154]. The reasons behind high false positives include reactive epithelial atypia and dysplasia, but recent advances, such as transepithelial brush biopsy, liquid biopsy, standardized reporting, and adjunct molecular analyses, have improved its specificity. Nevertheless, exfoliative cytology remains an adjunctive screening and triage tool, with histopathological analysis being a gold standard for OSCC diagnosis [155].

#### 8.1.4 Oral brush biopsy

The brush biopsy technique is a straightforward, non-invasive procedure performed in the dental chair that involves collecting samples from the entire thickness of the lesion. This technique uses a brush to perform a complete transepithelial biopsy, providing a cellular representation of all three layers of the lesion. To perform the biopsy, the brush is pressed against the lesion and rotated until small spots of bleeding are visible. The acquired cells are then mounted to a slide, coated, stained, and analyzed by a computer. Generally, this method has a sensitivity of 71.40% and a specificity ranging from 32.2% to 92.9% [150].

#### 8.1.5 Liquid-based cytology

Liquid-based cytology is one of the more recent advancements in screening techniques [150]. Here, samples are acquired by using a brush-like tool, which is then dipped into a vial containing a preservative solution. The sample is transported to the laboratory, where it undergoes a process to remove any materials that can obscure the view, such as mucus or blood. After clarification, the sample was centrifuged *via* standard methods to collect the cells. The liquid on top (the supernatant) is discarded, and the remaining cell layer is mixed with a special solution that helps create a clear film. This film was then carefully transferred onto a clean microscope slide. This approach allows for a cleaner and more accurate sample, with fewer background elements that can interfere with analysis [156], [157]. Research on liquid-based cytology has highlighted several benefits, including better sample preservation, improved adequacy, highly resolved cell structures, greater consistency, and reduced cell clumping [156], [158]. For example, a study by Hayama *et al.* [158] reported a 41% enhancement in smear thickness and a 66% improvement in how well the cells were spread across the slide.

#### 8.1.6 Fluorescence-based and optical biopsy technique

Fluorescence-based and optical biopsy techniques are becoming more popular in oncology research. Their growing popularity is largely due to their capacity to deliver quick, timely, and non-invasive tissue diagnostics directly at the site of concern [159], [160]. It is often integrated with Raman spectroscopy and elastic scattering spectroscopy (ESS) [150] for providing a better molecular basis of disease through multiparametric analyses, multispectral imaging, and overcoming sensitivity limitations [159]. Fluorescence spectroscopy enables the detection of endogenous fluorophores like NADH and FAD for metabolic reprogramming, tryptophan for detecting protein alterations, and collagen and elastin for assessing stromal degradation, prior to conventional oral morphological lesions observation [134]. Neoplastic cells exhibit minimal auto-fluorescence and spectral shift, while normal mucosal cells don’t. This chairside screening technique is not only invasive but also rapid and feasible for patients with low income [150], [160].

#### 8.1.7 Vizilite

Vizilite is a non-invasive screening tool based on the principle of chemiluminescence. It involves applying a CH_3_COOH solution, followed by the addition of a cytoplasmic drying agent, and then examining the tissue under a chemiluminescent light source. When this light is applied, healthy mucosal cells absorb blue-white light, while cells with dysplastic nuclei, such as neoplastic cells, reflect it. As a result, areas with dysplastic or neoplastic changes appear bright white, whereas normal tissue has a bluish hue [161]. The sensitivity of Vizilite in detecting neoplastic tissues has been reported to vary widely from 0% to 100%, while its specificity ranges from 14.2% to 81.5% [161], [162], [163]. Owing to this variability, the method’s main limitations are its low specificity and the high number of false positives. However, these issues can potentially be minimized by combining vizilite with toluidine blue staining [150], [163].

#### 8.1.8 VELscope

The VELscope is a compact diagnostic device that utilizes light emitting a spectral band of 400–460 nm to facilitate direct visualization of tissue fluorescence. Upon illumination, healthy oral mucosa exhibits green autofluorescence, whereas abnormal tissue absorbs fluorescent light, resulting in a darker, non-fluorescent area [163]. The sensitivity and specificity of this device have been reported to range from 97%–98% and 94%–100%, respectively [150], [163].

#### 8.1.9 Microfluidics lab-on-a-chip technology

Microfluidic lab-on-a-chip technologies are increasingly recognized as effective platforms for the non-invasive, rapid, and cost-efficient diagnosis of OSCC [164]. By integrating numerous laboratory processes, including sample preparation, analyte separation, and detection into a single, miniaturized device, these systems enable analysis from minimal saliva volumes with swift turnaround times, thus providing a significant advantage over conventional assays such as enzyme-linked immunosorbent assay (ELISA) or quantitative polymerase chain reaction (qPCR) [150]. A prominent example is the University of California, Los Angeles (UCLA) electrochemical microfluidic chip, which has demonstrated approximately 90% sensitivity and specificity in detecting the IL-8 protein and its mRNA in OSCC and shows performance comparable to that of traditional laboratory methods while offering point-of-care applicability [165]. Microfluidic lab-on-a-chip technology is capable of multiplexing, allowing for the simultaneous detection of proteins, nucleic acids, and metabolites, which is crucial given the biological heterogeneity associated with OSCC [166].

#### 8.1.10 Nanotechnology and nanomaterials

With the advent of nanotechnology, researchers have explored diverse nanomaterials for disease diagnosis. Nanomaterials, specifically gold-based nanoparticles (GNPs), have emerged as valuable instruments for the diagnosis of OSCC, leveraging distinct optical properties to enable sensitive and specific cancer detection, including applications in margin mapping *via* gold nanorods, non-invasive surface-enhanced Raman spectroscopy saliva assays, and photoacoustic detection of micrometastasis [167].

Gold nanorods (GNRs) have resolved one of the key challenges in the surgical management of OSCC, such as the precise identification of tumor margins, as incomplete resection considerably increases the risk of recurrence. Hence, GNRs were conjugated to anti-EGFR antibodies to delineate tumor boundaries. The high overexpression of EGFR in OSCC relative to normal epithelial cells renders it a promising ideal biomarker. This characteristic feature was exploited by Ankri *et al.* [167] who visualized EGFR-targeted GNRs in OSCC tissue sections *via* air-scanning electron microscopy and diffusion reflection (DR) imaging techniques. The results indicated a gradient of nanoparticle distribution extending up to 1 mm from the tumor into the adjacent normal epithelium, suggesting that GNR-based imaging can facilitate accurate tumor margin mapping during surgical procedures. This approach effectively addresses the limitations associated with frozen section analysis and magnetic resonance imaging, which are often compromised by artifacts and diminished resolution [167].

Similar application of colloidal gold nanoparticles has emerged, leveraging their surface plasmon resonance (SPR) properties. The study conducted by Kah *et al.* [168] demonstrated that spherical gold nanoparticles, approximately 15 nm in size, conjugated with anti-EGFR antibodies significantly improved confocal reflectance microscopy imaging of cancer cells. These nanoparticles increased the scattering intensity, thereby creating a pronounced optical contrast between cancerous and normal cells. Notably, compared with normal fibroblasts, EGFR-targeted gold nanoparticles selectively accumulated in OSCC cells, resulting in up to 30-fold greater reflectance. This selectivity enables molecular-level mapping of EGFR expression, thus providing a non-invasive and highly specific diagnostic modality. Furthermore, SPR-induced scattering facilitates detection at lower concentrations of nanoparticles, thereby enhancing diagnostic sensitivity [168].

The diagnosis of OSCC necessitates a precise assessment of lymph node involvement, as this significantly impacts prognosis. Traditional sentinel lymph node biopsy (SLNB) is considered both invasive and limited in sensitivity [169]. In 2014, Luke *et al.* [170] introduced molecularly activated plasmonic nanosensors (MAPSs), which are composed of 40 nm gold nanoparticles conjugated with anti-EGFR antibodies and polyethene glycol. By employing ultrasound-guided spectroscopic photoacoustic (sPA) imaging, these nanosensors demonstrated the ability to detect micrometastases as small as 50 μm within murine models of OSCC. Importantly, metastases were identifiable within 30 min following the injection of MAPS, thus presenting a rapid and non-invasive alternative to SLNB. This innovative technique effectively combines the depth of ultrasound penetration with the molecular specificity of plasmonic nanoparticles, establishing its importance for the clinical staging of OSCC [170].

### 8.2 Invasive oral cancer screening techniques

#### 8.2.1 Fine needle aspiration cytology (FNAC)

Research conducted by Seetharam *et al.* [171] highlighted that FNAC is a dependable diagnostic tool for OSCC, although it offers limited diagnostic value in cases of oral leucoplakia. Several other researchers have also explored the use of FNAC for assessing tumors and lesions in the oral cavity, oropharynx, nasopharynx, and maxillary regions. Most of these investigations have reported favorable outcomes, particularly in diagnosing SCC [150], [172].

#### 8.2.2 Biopsy and histopathology

Even with the wide range of diagnostic tools available for detecting cancer, biopsy followed by histopathological analysis is still considered the most reliable method [157], [173]. While clinical tools can assist in visualizing a lesion, only histopathology can accurately determine how deep, extensive, or severe it is [150], [157]. The transition from a precancerous state to full-blown cancer has been linked through histological studies to a stepwise progression initiating with epithelial dysplasia, then progressing to in situ neoplasm, and eventually developing into carcinoma [174].

### 8.3 Molecular methods

A wide range of molecular tools is now available to explore the changes that occur in the TME. Molecular methods can be used for both invasive and non-invasive samples. Molecular techniques for oral cancer screening include several hybridization-based techniques, such as PCR, fluorescent in situ hybridization (FISH), microarrays, flow cytometry, immunohistochemistry (IHC), tumor marker detection, and laser capture microdissection (LCM). They help in understanding tumor biology, diagnosing cancer at the cytogenetic level, mapping chromosomes, validating prognostic and molecular tumor markers, discovering new biomarkers, determining DNA ploidy, and carrying out immunophenotyping [174].

Among all molecular techniques utilized for oral cancer detection, PCR has become a cornerstone in cancer research. It is especially useful when working with small quantities of unique DNA sequences and has been successfully applied in detecting cancerous cells in bodily fluids such as urine, sputum, and saliva [174]. Additionally, FISH allows the detection of two or more genetic targets at once, whereas microarrays are powerful tools for tumor analysis and are capable of assessing the expression profiles of several genes in real time through multiplexing approaches [175]. Flow cytometry offers a rapid and efficient way to measure DNA content in isolated cells or nuclei by analyzing their fluorescence levels [176]. Both IHC and tumor markers have been extensively investigated, covering a broad spectrum of biological factors, such as TSGs, proto-oncogenes, growth and angiogenic factors, cell adhesion molecules, and markers related to tumor invasion, metastasis, loss of heterozygosity (LOH), and DNA aneuploidy [174].

Along with the growing emphasis on deciphering the genetics of cancer, a corresponding surge in the evolution of advanced molecular techniques has occurred. These advances will greatly improve diagnostic accuracy and enhance patient palliative care plans in the future [150], [177].

## 9 Salivary liquid biopsy in OSCC: physiological, biochemical, and standardization considerations

To reduce mortality associated with OSCC, the most urgent and effective approach lies in improving and advancing technologies for early OSCC diagnosis and detection. Among these methods, liquid biopsy has surfaced as a promising, non-invasive diagnostic method that works by identifying tumor markers present in body fluids [178], [179], [180]. Remarkably, it enables the detection of both genetic and epigenetic alterations, involving changes in gene and epigenomic expression profiles through a range of biomarkers [178], [179], [180], [181]. These include CTCs,ctDNA, miRNAs, and extracellular vesicles (EVs), all of which can be found in fluids such as saliva, blood, serum, plasma, and pleural fluid, and urine [178], [179].

One major benefit of the assessment of cancer biomarkers *via* liquid biopsy is its ability to provide an instantaneous representation of primary and metastatic tumors at different time points. In addition to serving as an aid in detecting the presence and size of a tumor, this method also picks up early signs of drug resistance and potential recurrence [182]. Furthermore, analysis of patients’ DNA in liquid biopsy samples can lead to the integration of molecular information into the TNM system. This integration will spur the use of increasingly personalized and targeted therapeutic approaches and reduce the risk of implementing unnecessary or ineffective interventions [183].

### 9.1 Salivary physiology and biochemical composition

Saliva is a clear, odorless, and hypotonic solution composed of various secretions from salivary acini, gingival crevicular fluid, and exudates from the buccal mucosa. It has a relative density ranging from 1.002 to 1.012 g/ml. Approximately 90% of saliva production originates from the acinar cells found within the salivary glands [184]. Saliva plays several vital roles: it aids digestion by moistening and softening food, facilitates chewing and swallowing, helps maintain the oral pH within a range of 6.6–7.1, promotes oral hygiene, and offers immune protection against various microbial threats [184].

The salivary makeup is complex and diverse, containing a mixture of biochemical substances such as urea, ammonia, uric acid, glucose, cholesterol, fatty acids, triglycerides, neutral lipids, glycolipids, and amino acids. It also includes steroid hormones, enzymes (such as amylase and peroxidase), mucins, glycoproteins, and a variety of peptides (such as defensins, calprotectin, adrenomedullin, histatins, cystatins, and lactoferrin). Additionally, saliva contains high levels of electrolytes such as Na⁺, Cl⁻, Ca²⁺, and many more, primarily derived from serum.

Salivary glands are richly vascularized and highly permeable, which permits the exchange of a wide array of molecules, including DNA, RNA, proteins, and microbial components. This intricate mixture makes saliva a valuable diagnostic medium. The field of “salivaomics” has emerged to harness this potential, enabling the identification of biomarkers for various diseases [185]. Saliva acquisition is non-invasive and generally safe, making it a promising alternative to more invasive diagnostic procedures such as blood draws or tissue biopsies. Several proteomic, transcriptomic, and microbiological markers identified in saliva show strong potential for disease detection, thus supporting its growing use in diagnostics [184].

With the discovery of numerous molecular markers in saliva that are associated with both OSCC and other diseases, saliva-based diagnostics are gaining traction [186], [187], [188].

### 9.2 Preanalytical and analytical standardization of saliva

The complex composition of saliva and its susceptibility to environmental factors necessitate stringent standardization at both the preanalytical and analytical stages. This includes established protocols for the collection, handling, storage, transportation, and selection of diagnostic assays. In the absence of such management, variability in results may arise, ultimately limiting reproducibility and clinical applicability. Certain preanalytical and analytical standards need to be considered.

#### 9.2.1 Preanalytical standardization

Saliva collection represents the most critical preanalytical variable, as it directly influences the concentration and integrity of biomarkers. In research and diagnostic contexts, both unstimulated and stimulated saliva are utilized; however, unstimulated whole-mouth saliva is typically the preferred option by clinicians and researchers for baseline measurements owing to its reduced variability [189], [190]. Participants are usually instructed to refrain from consuming food and beverages, smoking, or engaging in oral hygiene practices for at least 90 min before saliva collection to minimize the risk of contamination and compositional fluctuations. Furthermore, circadian influences necessitate standardized collection times, which are commonly scheduled between 9:00 a.m. and 11:00 a.m., a period during which salivary flow and hormone levels are relatively stable [189], [190], [191]. Additionally, the method of collection warrants careful attention. Passive drooling into sterile polypropylene tubes is considered the gold standard because of its minimal risk of contamination and interference [189], [190], [191]. Commercial devices, such as Salivette® swabs, can facilitate sampling, particularly in pediatric or geriatric populations; however, these devices may adsorb proteins and introduce variability. Importantly, the collection of stimulated saliva through the use of citric acid or chewing gum is discouraged in proteomic and transcriptomic analyses, as stimulation can alter the ionic content, protein concentration, and enzymatic activity [189], [190].

Upon collection, saliva is susceptible to enzymatic degradation by endogenous proteases and RNAses, as well as bacterial activity. To mitigate these risks, samples are subjected to centrifugation at approximately 3000 r/min for 15 min at 4 ℃, effectively removing debris, cells, and microorganisms. The clarified supernatant is subsequently aliquoted into cryovials to prevent degradation resulting from repeated freeze-thaw cycles. Depending on the analytical objectives, protease or RNase inhibitors may be added at this stage. For example, RNA-based studies typically incorporate the utilization of RNA later or similar stabilizing agents to preserve transcript integrity [189].

Short-term storage at 4 ℃ is permissible for a duration of up to 24 h; however, this condition is primarily appropriate for protein assays. Importantly, nucleic acids experience rapid degradation at this temperature, rendering them unsuitable for analyses involving RNA or DNA. Different stability studies of biomarkers, such as inflammatory cytokines, such as IL-6 and C-reactive protein (CRP). However, for long-term preservation, a temperature of −80 ℃ is considered the standard. At this specific temperature, proteins, nucleic acids, and metabolites can maintain their stability for extended periods, allowing for longitudinal studies and effective biobanking. The stability profiles presented in different studies demonstrate that both DNA and proteins exhibit minimal degradation after 6 months at −80 ℃, whereas RNA preservation is optimized through the addition of stabilizing agents. To mitigate the effects of repeated thawing, it is strongly advised to aliquot samples into single-use vials, as multiple freeze-thaw cycles can lead to significant loss of protein integrity and result in RNA fragmentation [189].

Transport serves as a vital connection between sample collection and laboratory analysis, particularly in decentralized or field-based research studies. Ideally, samples should be transported on dry ice to maintain a stable temperature of −80 ℃, after which they must be transferred to ultralow freezers. Furthermore, saliva collected in commercial stabilizing buffers demonstrates prolonged stability, thereby facilitating transportation from remote locations without the need for immediate freezing. It is essential to adhere to these guidelines to ensure the reliability of diagnostic results [189].

#### 9.2.2 Analytical standardization

Salivary biomarkers hold excessive potential for facilitating the diagnosis and monitoring of this disease without the need for invasive procedures. Hence, a range of analytical methods is employed in saliva-based diagnostics to detect alterations, quantify biomarkers, and standardize the analysis. Commonly used methods include LC-MS, ELISA, PCR, immunoblotting, two-dimensional gel electrophoresis (2D-GE), various chromatographic approaches, and nuclear magnetic resonance (NMR), which are listed in Table 3 [36], [192], [193], [194], [195], [196], [197], [198], [199], [200], [201].

**Table 3:** Different analytical techniques employed to identify different salivary biomarkers in the saliva of OSCC patients.

**Analytical methods**	**Application**	**Identification of salivary molecule**	**References**
ELISA	Non-invasive and point-of-care testing allowing for the sensitive and specific quantification of salivary biomarkers	EGF, Ki-67, p53, PDL1, HLA-E, B7-H6, MMP-1, IL-6/-8/-1β, CD44	[192]
qPCR	Non-invasive and highly sensitive technique allowing for amplification of the specific DNA and mRNA sequences, and detection of the mutant genetic markers that may be present in low quantities in saliva in real time.	*miR-125a*, *miR-200a*, *miR-31*, *miR-412-3p*, *IL-8*, *IL1B*, *DUSP1*, *HA3*, *OAZ1*, *S100P*, and *SAT*	[193], [194]
Immunoblotting	Highly specific technique useful for protein identification and quantification, and allowing for the post-translational modifications detections	IL-1α/-1β/-6, TNF-α, VEGF-A, MMP-1/3/9, EGF, Ki-67, Cyclin D1, GSK3β, cadherins, FGF, survivin, STAT3.	[36], [195], [196]
Mass spectrometry	Aids in oral cancer detection by differential expression analysis and quantification of the unique molecular signatures (proteins, metabolites, lipids) present in oral fluids, helping to distinguish between healthy and cancerous tissues	CFH, FGA, and SERPINA1	[197]
Chromatography	Helps to separate and analyze the complex mixture of molecules in saliva, allowing identification of specific markers and their molecular weight through coupled mass spectrometry	M2BP, MRP14, CD59, catalase, profilin, MMP1/3/9	[198], [199]
NMR	Aids in analyzing the metabolic changes in the saliva of OSCC patients compared to control individuals	Butyrate, propionate, lactate, alanine, butanol, acetate, pyruvate, succinate, methylamine, choline, taurine, methanol, proline-tyrosine, phenylalanine, formate, glycine, 1,2-propanediol, fucose	[200]
2D-GE electrophoresis	Salivary protein separation based on their isoelectric point and molecular weight, resulting in a high-resolution separation of proteins, allowing for the identification of subtle differences in protein expression	Serum albumin, Hsp27, SCC1, ANX4, γ-actin	[201]

ELISA. Enzyme-linked immunosorbent assay; qPCR. Quantitative polymerase chain reaction; NMR. Nuclear magnetic resonance; EGF. Epidermal growth factor; Ki-67. Kiel-67; p53. Tumor protein 53; PD-L1. Programmed death-ligand 1; HLAE. Human leukocyte antigen E; B7H6. Natural cytotoxicity receptor 3 ligand 1; MMP. Matrix metalloproteinase; IL. Interleukin; CD44. Cluster of differentiation 44; DUSP1. Dual specificity phosphatase 1; HA3. Hyaluronan Synthase 3; OAZ1. Ornithine decarboxylase antizyme 1; S100P. S100 calcium-binding protein P; SAT. Serine acetyltransferase; TNF-α. Tumor necrosis factor-α; VEGFA. Vascular endothelial growth factor A; GSK3β. Glycogen synthase kinase 3 beta; FGF. Fibroblast growth factor; STAT3. Signal transducer and activator of transcription 3; CFH. Complement factor H; FGA. Fibrinogen alpha chain; SERPINA1. Serpin family member 1; M2BP. Mac2 binding protein; MRP14. Myeloid-related protein 14; Hsp27. Heat shock protein 27; miR. microRNA; SCC1. Sister Chromatid Cohesion protein 1; ANX4. Annexin A4; **γ-**actin. Gamma actin; 2D-GE. Two-dimensional gel electrophoresis

***Immunoassays** T*he ELISA technique is widely regarded as the predominant method for salivary diagnostics owing to its exceptional specificity, adaptability to a diverse array of biomarkers in 96-well formats, and multiplexing [189], [192]. Standardized ELISA kits facilitate the reliable quantification of proteins such as cortisol, CRP, and cytokines [192]. Additionally, multiplex bead-based immunoassays enhance testing efficiency by enabling the concurrent detection of multiple analytes from small saliva aliquots, thereby conserving biological materials [189]. Jaedicke *et al.* [202] analyzed saliva samples ranging from 5 to 10 ml obtained from healthy volunteers to perform multiple ELISA experiments, including assays for CRP, hepatocyte growth factor (HGF), IL-1β, IL-6, MMP-3, MMP-8, MMP-9, Rantes, and Tissue Inhibitor Of Metalloproteinase 1 (TIMP-1). Upon immediate collection, the saliva samples must be stored in a −80°C freezer prior to analysis. During the assay preparation phase, samples are retrieved from the −80°C freezer and rapidly thawed in a water bath set at 37°C, after which the ELISA is conducted in accordance with the manufacturer’s protocols. Notably, the majority of ELISA methods demonstrate reliable performance with saliva, even when the assay was not specifically designed for this biological matrix [192]. However, it is particularly well-suited for screening and early diagnostic purposes, especially when targets, such as the interleukins IL-6, IL-8, and IL-1β, as well as CD44, have been prevalidated. Its strengths include high analytical sensitivity and specificity, in addition to straightforward handling of saliva samples. However, it is dependent on the availability of high-quality antibodies and has a limited panel of detectable analytes [192].

***Molecular techniques*** PCR and reverse transcription-PCR (RT-PCR) are recognized as the gold standards for the detection of nucleic acids in saliva. For example, an investigation was conducted regarding the standardization of salivary DNA and RNA as case-control experimental studies aimed at validating saliva as a non-invasive medium for the diagnosis of OSCC [203]. For standardization, unstimulated saliva samples were collected from 45 individuals: healthy (*n*=15), dysplasia patients (*n*=15), and patients with advanced metastatic neoplasms (*n*=15). Nucleic acid samples obtained from these samples were subjected to PCR analysis, which revealed optimal results, confirming that proper preanalytical alterations can be easily detected in OSCC [76], [203]. qPCR is a more sophisticated and sensitive technique than conventional PCR, as it offers very high-throughput capacity using 96- or 384-well formats and provides excellent sensitivity for detecting low-abundance nucleic acids [76]. This makes it particularly effective for the early identification of microRNAs, such as miR-125a, *miR-200a*, *miR-31*, and *miR-412-3p*, as well as messenger RNAs, including *IL-8*, *IL1B*, *DUSP1*, *HA3*, *OAZ1*, and *SAT* [193]. While qPCR is powerful for early diagnosis and requires minimal sample input, it encounters challenges related to preanalytical RNA integrity and the standardization of assay conditions [193], [194].

***Proteomics and metabolomics*** Both discovery and targeted proteomics *via* mass spectrometry provide moderate- to high-throughput methods with extensive coverage, which facilitates the discovery of panels at early stages and the quantitative validation of analytes such as complement factor H (CFH), fibrinogen alpha chain (FGA), and serpin family a member 1 (SERPINA1) [197]. Mass spectrometry excels in its ability to perform multiplexing and objective quantitation; however, it necessitates specialized infrastructure and rigorous quality control measures for effective clinical translation [197]. Mass spectrometry (MS), particularly LC-MS/MS, has significantly advanced the field of salivary proteomics by enabling comprehensive profiling of proteins, peptides, and metabolites. Chromatographic techniques contribute to enhanced selectivity and support panel-level assessments of various biomarkers. Mac2 binding protein (M2BP), myeloid-related protein 14 (MRP14), CD59, catalase, profilin, and MMP-1, MMP-3, MMP-9 are certain examples that have been assessed through chromatographic techniques [197], [199]. While the throughput is moderate, chromatography demonstrates strong applicability for early diagnostic purposes when utilized within targeted workflows. Nevertheless, the development and validation of methodologies in this context can be complex [199].

Costa *et al.* [204] collected saliva from different acquisition methods to evaluate the sample variability based on MS-proteome profiling. Their study revealed that unstimulated saliva samples produced the most stable proteomic profiles, while stimulated ones showed high variability of altered proteins and mucins. In contrast, gland specific saliva sample couldn’t be processed properly due to limited sample volume and inherent variability. This study underscored that sample collections need to be standardized pre-analytically for better outcomes, reliable, and reproducible salivary proteomic data. Gardner *et al.* [205] conducted NMR-based salivary metabolomics to evaluate the impact of metabolomics on different saliva collection methods, for which he concluded with similar results.

***Electrophoretic techniques*** Electrophoretic techniques play a critical role in salivary biomarker research by enabling researchers to separate, visualize, and compare proteins derived from diseased patients and healthy controls [36], [195]. Electrophoretic techniques exploit both one-and two-dimensional approaches [196]. 2D-GE and western blotting are two key techniques that allow researchers to discover and validate the disease-associated salivary biomarkers [201]. 2D-GE allows clinicians to evaluate the protein bands on the basis of isoelectric point and molecular weight, while western blotting validates and strengthens those proteomic findings at preclinical and clinical cohorts [196], [201]. 2D-GE facilitates high-resolution separation of specific proteins [such as serum albumin, Hsp27, SCC1, annexin A4 (ANX4), and γ-actin], but it necessitates MS analysis [201].

## 10 Salivary biomarkers associated with OSCC

Research has identified numerous salivary biomarkers across different molecular classes that are associated with OSCC, which holds significant promise for prompt detection, diagnosis, prognosis, and assessment of OSCC.

### 10.1 ctDNA

The presence of DNA fragments in human plasma was first identified in 1948 by French scientists Mandel and Métais [206], who described them as cell-free deoxyribonucleic acid (cfDNA). This discovery later paved the way for the recognition of ctDNA, which consists of single- or double-stranded DNA fragments originating from tumors and entering the bloodstream. Fragmentized DNA is usually released by neoplastic cells, either when the cells die naturally or when they actively release tiny vesicles that contain DNA [181]. While there are different notions about how this happens, we still do not fully understand it. Generally, ctDNA fragments are shorter than normal cell-free DNA, but there are still no clear details about their exact size. Nevertheless, ctDNA has received much attention because it could be useful for screening for cancer, managing treatment, and tracking the disease [181], [207], [208].

ctDNA reflects both physiological and pathological processes, and carries genetic and epigenetic markers unique to tumor cells. It typically accounts for less than 1.0% of total cfDNA, yet it serves as a powerful biological marker for prompt cancer diagnosis and real-time tracking of tumor behavior [207], [209]. Additionally, the levels of ctDNA are driven by various tumor-related factors, including size, cell turnover, disease stage, vascular supply, and response to treatment. The current diagnostic status of ctDNA in the OSCC population is described in Table 4 [210], [211], [212], [213].

**Table 4:** Diagnostic status of ctDNA in OSCC patients on the basis of population studies.

**Patient cohort**	**Age (years)**	**Sample type**	**HPV/p16 status**	**Control group information**	**Biomarker utility**	**Normalization/** **Standardization**	**Limitations**	**References**
77 (64 male/13 female)	42–84	Blood&Saliva ctDNA	60 (+)/17 (–)	HPV-group served as control	Over 90% samples reported ctDNA positive (pretreatment), but post-treatment, only 9% samples reported positive ctDNA count. High correlation (>90%) was observed between saliva and blood ctDNA sampling	No explicit cfDNA load or internal control normalization was included; also, positivity thresholds were not harmonized	Small HPV- control with no standardized assay reported	[210]
100/116 (79 male/37 female)	21–94	Tumor tissue&Blood	21 (HPV&p16+ve); 44 (p16/HPV–ve) and 51 (not tested)	No defined healthy control; HPV/p16-groups used for comparison	ctDNA was well detected in prior treatment as compared to post-treatment, 75% and 25% samples were positively and negatively detected, respectively, with ctDNA prior to treatment	Tumor-informed sequencing improves specificity. Explicit normalization to cfDNA is not reported	High proportion of untested HPV status; Limited cross-study standardization	[211]
37 (NA)	18–70	Tumor tissue, plasma, and oral rinses	HPV–ve	HPV– cohort (No HPV+ available)	TP53 mutations were detected in plasma & oral rinses; however, oral rinses had higher incidences of TP53 mutations	Compared methods; No uniform normalization as the cfDNA background is not standardized	HPV^–^ samples only; Reduced generalizability	[212]
37 (31 male/6 female)	22–81	Saliva	NA	No clear control group	Genetic alterations observed in the HNSCC samples (in several regions of p-chromosomal arms of chr3, 6, 9, 17, and q-chromosomal arms of chr4, 5, 11, 13, 18, 20)	Exploratory; No normalization framework reported	Early stage design; Weak statistical robustness	[213]

HPV. Human papillomavirus; ctDNA. Circulating tumor deoxyribonucleic acid; cfDNA. Cell-free deoxyribonucleic acid; TP53. Tumor protein 53; chr. Chromosome‌; NA. Not available

The comparative evaluation of ctDNA studies in OSCC presents promising evidence for its utility as a non-invasive biomarker for diagnosis, treatment monitoring, and recurrence prediction. Ferrier *et al.* [210] demonstrated a strong correlation between ctDNA levels in saliva and blood, as well as a significant reduction in ctDNA levels posttreatment, underscoring its effectiveness in monitoring residual disease. However, this study faced limitations due to the small sample size of the HPV-control group [180]. Similarly, Hanna *et al.* [211] highlighted the benefits of personalized, tumor-informed ctDNA monitoring, although the lack of comprehensive HPV/p16 testing weakened the comparability of their cohort. Additionally, Perdomo *et al.* [212] reported the presence of TP53 mutations in oral rinses and plasma exclusively in HPV-patients, thereby restricting their general applicability. Sethi *et al.* [213] offered preliminary exploratory insights into chromosomal alterations in saliva but did not use rigorous normalization or statistical standardization. A study using a mouse model of OSCC induced by FaDu cells revealed that, before surgery, saliva samples contained higher concentrations of long interspersed nuclear element-1 (LINE-1) than did the tumor tissue itself. However, following surgical removal, LINE-1 levels in saliva significantly decrease, and unlike saliva samples, ctDNA in tissue samples does not significantly change [214].

A standard limitation across these studies is the absence of standardized normalization frameworks, such as adjustments for cfDNA yield, tumor fraction, or background noise, which hinders reproducibility and consistency across studies. Therefore, while ctDNA shows significant potential as a clinical biomarker, the establishment of standardized preanalytical processes, normalization methods, and HPV/p16 stratification is imperative for its effective implementation in clinical practice.

### 10.2 Exosomes and miRNAs

Exosomes, a specific category of EVs ranging in size from 30 to 150 nanometers, are produced through a tightly regulated endosomal pathway. These nanoscale vesicular components play crucial roles in facilitating cell-to-cell communication within the TME of OSCC and have been implicated in processes such as tumor initiation, local invasion, metastasis, immune evasion, and resistance to therapy [215].

The formation of exosomes commences with the inward budding of the cell membrane, directing the production of early endosomes (Ees). These EEs then mature into late endosomal vesicles, which further develop into multivesicular bodies (MVBs) through the intracellular budding of their cell membranes. This process produces intraluminal vesicles (ILVs) within MVBs, which eventually become exosomes. ILVs selectively incorporate various bioactive molecules, including proteins such as the tetraspanins CD63, CD81, and CD9; lipids; messenger RNAs; miRNAs such as miR-21 and miR-155; and long non-coding RNAs. The sorting of these molecular components is orchestrated through mechanisms involving the endosomal sorting complexes required for transport (ESCRT) system or *via* ESCRT-independent pathways involving molecules such as tetraspanins and ceramides [216]. Following their formation, MVBs have two potential fates: they may be directed toward lysosomal degradation, or they may fuse with the cell membrane, releasing their ILV contents into the extracellular space as exosomes. In the context of OSCC, tumor-derived exosomes are particularly enriched with oncogenic cargo that mirrors the molecular profile of their originating tumor cells. These exosomes can profoundly influence neighboring and distant recipient cells by driving EMT, promoting angiogenesis, modulating immune responses, and facilitating remodeling of the ECM [216].

In recent years, miRNAs, which are short, single-stranded non-coding RNA molecules approximately 20 nt in length, have gained attention as valuable biomarkers for OSCC detection, prognosis, and therapy. Owing to their notable stability in saliva, miRNAs are particularly suitable for non-invasive diagnostic applications. Functionally, miRNAs regulate the expression profile by binding to the 3’UTRs of target mRNAs, thereby promoting mRNA degradation or inhibiting translation [217]. A focused study in South Indian patients examined the expression of miR-21 in OSCC tissues. Using bioinformatics tools and q-PCR, researchers reported marked upregulation of miR-21 in tumor tissues compared with adjacent normal regional tissues. This upregulation profile was significantly linked with advanced OSCC clinical stages, necessitating its potential role as a prognostic marker. These findings suggest that miR-21 may serve as a predictive biological marker for OSCC progression and could facilitate early diagnosis and the development of RNA-based therapeutic interventions targeting this specific miRNA [70].

In another comprehensive profiling study, researchers analyzed the expression profiles of more than 1100 miRNAs in OSCC tissues from Indian patients to explore the molecular mechanisms underlying tumorigenesis. They identified 46 differentially expressed miRNAs, including downregulated members of the let-7 family (let-7a/7d/7 f) and miR-16, as well as upregulated miR-29b, miR-142-3p, miR-144, miR-203, and miR-223. Importantly, miR-1275 was reported to be associated with lymph node metastasis in OSCC. Subsequent pathway analysis indicated that these deregulated miRNAs may contribute to carcinogenesis by triggering the PI3K/Akt/mTOR pathway, through the suppression of tumor-suppressor miRNAs, and inhibiting the tumor-suppressor p53 pathway *via* the overexpression of oncogenic miRNAs. These results emphasize the promise of miRNAs as detection and predictive indicators, as well as potential OSCC therapeutic targets [218].

Additionally, miR-196a is notably amplified in OSCC cells relative to normal oral epithelial cells. This miRNA appears to act as an oncogene by enhancing cellular proliferation and migration while inhibiting programmed cell death (apoptosis). Mechanistically, miR-196a exerts these effects through the negative regulation of the *FOXO1* gene and the modulation of the PI3K/Akt/mTOR pathway. Therefore, therapeutic strategies aimed at inhibiting miR-196a or disrupting the miR-196a/FOXO1 axis are proposed as novel approaches for treating OSCC [219].

Numerous miRNAs have been identified within exosomes derived from the saliva, serum, and plasma samples of OSCC patients, as listed in Table 5 [220], [221], [222], [223], [224], [225], [226], [227]. Notably, miR-486-5p is significantly upregulated in the saliva of OSCC patients, particularly individuals diagnosed with stage II tumors. Faur *et al.* [190] reported a considerable increase in the fold change compared with that in control groups, emphasizing its potential as a robust biomarker. The upregulation achieved statistical significance, thereby reinforcing its applicability for diagnostic purposes [190]. In contrast, miR-10b-5p was downregulated in the same patient cohort; however, this decrease did not achieve statistical significance, suggesting variability in its function across various cancer types [220], [221].

**Table 5:** Exosomal cargos carrying different miRNAs in the serum, plasma, and saliva samples of the investigated OSCC patients.

**OSCC biomarkers**	**Expression**	**Results**	**Fold change compared to control**	**References**
miR-486-5p	Upregulated	Highly upregulated in Stage II cancer	A 3- to 4-fold increase is observed with the advancement of each OSCC stage, with statistical significance (*P*<0.05)	[220], [221]
miR-10b-5p	Downregulated	Insignificant	A 1.5- to 2-fold decrease was observed compared to controls, not statistically significant, and variability across cohorts	[220], [221]
miR-24-3p	Upregulated	Upregulation of miRNA-24-3p promoted the OSCC proliferation through dysregulation of cell cycle-regulatory genes	5-fold increase in salivary exosomes of OSCC patients relative to healthy controls, with statistical significance (*P*<0.01)	[222]
miR-1307-5p	Upregulated	Promotes OSCC by suppressing onco-related genes THOP1, EHF, RNF4, GET4 and RNF114; Reported to be associated with poor prognosis	An approximately 6-fold increase was observed in OSCC patients compared to healthy controls (*P*<0.05)	[223]
miR-134/200a, IL-1β, and IL-8	Upregulated	No significant changes were observed between different OSCC grades	Approximately 2- to 3-fold increase in OSCC saliva samples as compared to controls, with no statistical significance	[224]
**Exosomal cargos identified in OSCC serum samples**				
miR-155/21	Upregulated	Downregulates tumor suppressors such as PTEN and Bcl-6	3- to 5-fold increase in circulating exosomal miRNA 155/21 in serum with significance (*P*<0.05)	[225]
**Exosomal cargos identified in OSCC plasma samples**				
miR-130a	Upregulated	Linked with poor prognosis and advanced TNM staging and grading	4-fold change in plasma exosomes of OSCC patients relative to healthy controls, exhibiting statistical significance (*P*<0.01)	[226]
TGF-β	upregulated	Oncogenic role with advancing tumors	Elevated levels of TGF-β-bearing exosomes have a 2-fold change in advanced OSCC stages with significance (*P*<0.05)	[227]

THOP1. Thimet oligopeptidase 1; EHF. ETS Homologous factor; RNF4. RING Finger protein 4; GET4. Guided entry of tail-anchored protein factor 4; RNF114. RING Finger protein 114; PTEN. Phosphatase and tensin homolog; Bcl-6. B-cell lymphoma 6; TNM. Tumor node metastasis

Another promising candidate is miR-24-3p, which is significantly upregulated in the salivary exosomes of OSCC patients. He *et al.* [222] demonstrated that its upregulation facilitated OSCC proliferation by disrupting the expression of cell cycle regulatory genes. The reported fold change was statistically significant, positioning miR-24-3p as a potential biomarker for early screening [222]. Similarly, miR-1307-5p was also significantly upregulated in the saliva of OSCC patients, with Patel *et al.* [223] reporting that increased expression was associated with the suppression of tumor-regulatory genes [thimet oligopeptidase 1 (*THOP1*), epithelial height transcription factor (*EHF*), ring finger protein 4 (*RNF4*), guided entry of tail-anchored proteins 4 (*GET4*), and *RNF114*]. Its expression was strongly correlated with poor prognosis, highlighting its diagnostic and prognostic relevance [223].

The combined evaluation of miR-134 and miR-200a indicated that the expression of these genes was upregulated in OSCC salivary exosomes; however, Farag *et al.* [224] reported no significant changes when the data were stratified across different stages of OSCC. This observation suggests that while expression trends may be increasing, the statistical robustness is insufficient, emphasizing the need for validation in larger sample sizes [224]. Among serum-derived exosomes, miR-155 and miR-21 were significantly upregulated, which occurred through the downregulation of tumor suppressor genes such as PTEN and B-cell lymphoma 6 (Bcl-6), as noted by Chen *et al.* [225]. These findings align with characteristics associated with aggressive disease [224], [225].

Moreover, plasma-derived exosomal miR-130a was identified as significantly upregulated, with He *et al.*[226] linking its elevated expression to advanced TNM stage and poor prognosis. Finally, exosomes containing TGF-β were found to be elevated in the plasma of OSCC patients, as documented by Ludwig *et al.* [227], indicating that significant fold changes correlated with disease progression and reinforcing their role as indicators of tumor advancement. Overall, miR-486-5p, miR-24-3p, miR-1307-5p, miR-155, miR-21, and miR-130a [228] are characterized by statistically significant upregulation, with considerable fold changes, thus establishing their viability as biomarkers for OSCC. Conversely, miR-10b-5p, miR-134, and miR-200a, while altered, either lack statistical significance or exhibit inconsistency across different tumor grades. Additionally, the TGF-β exosomal cargo achieved statistical significance, contributing to the existing repertoire of prognostic biomarkers [227].

Recent studies have demonstrated that the cargo of exosome vesicles encompasses functional metabolites capable of reprogramming recipient cells [228], [229], [230]. In OSCC models, EVs derived from tumors alter the metabolic profile of normal oral fibroblasts within a period of hours to days, modifying pathways related to energy, amino acid, and lipid metabolism. This evidence suggests that the metabolite payloads are instructive rather than merely incidental. Comprehensive metabolomic analyses of both saliva and plasma in OSCC consistently reveal disturbances in ketogenesis, lipogenesis, and glycolysis, thereby supporting the concept of a metabolite-driven reconfiguration of the microenvironment and providing a rationale for the use of saliva as a source for metabolic biomarkers [229].

Growth factor cargo also enhances malignant characteristics in cancerous cells. The TGF-β protein encapsulated within OSCC EVs promotes EMT and facilitates prometastatic remodeling, thereby establishing a connection between vesicle signaling, EMT, and angiogenic alterations. Exosomal amphiregulin (AREG), a stable ligand for EGFR within cancer exosomes, amplifies invasion and survival signaling pathways [65], [66]. While a significant portion of the existing data are derived from studies involving other tumor types, the activation of the EGFR axis has emerged as a critical driver in OSCC. Consequently, AREG-positive exosomes represent a plausible factor contributing to both invasive behavior and drug resistance in oral tumors [229], [231].

miRNA cargo represents a significant regulatory mechanism. Exosomal miR-21, which has been frequently reported in OSCC, facilitates cisplatin resistance by repressing PTEN/PDCD4, thereby increasing cell survival and enabling evasion of therapeutic interventions [229]. Exosomes are also implicated in immune evasion mechanisms. In the context of HNSCC, including OSCC, circulating exosomal PD-L1 levels have been shown to correlate with unfavorable outcomes following radiotherapy and contribute to systemic T-cell suppression, which is a mechanism underlying resistance to immunotherapy in certain patient subgroups [229], [231]. From a translational research perspective, this framework supports two principal avenues: 1) the development of saliva-based exosomal panels for minimally invasive screening and monitoring, and 2) the formulation of therapeutics aimed at disrupting exosome biogenesis or uptake (for example, through targeting the Rab27 pathway) or neutralizing specific exosomal cargos (such as anti-TGF-β/EGFR and anti-PD-L1) to attenuate epithelial-mesenchymal transition, stromal reprogramming, and immune suppression [229], [231], [232].

Collectively, these findings underscore the importance of identifying distinct salivary exosomes and miRNA expression profiles associated with OSCC. These biomarkers could provide a critical understanding of disease pathophysiology and aid in the development of cost-effective, non-invasive screening tools. Ultimately, this could lead to enhanced patient outcomes through early diagnosis and more personalized therapeutics.

### 10.3 Circulating tumor cells

CTCs, which are malignant cells that break away from metastatic tumors and enter the bloodstream, play a pivotal role in neoplasm metastasis. Studying CTCs offers valuable opportunities for early diagnosis, prognostication, real-time monitoring of disease dynamics, and tailoring of therapeutic strategies [233].

These EMT-altered cells can then infiltrate blood vessels and circulate in the bloodstream, surviving despite immune attacks and mechanical stress. CTCs may exist as solitary cells or as aggregates called circulating tumor microemboli, which display greater resistance to apoptosis and possess a greater metastatic capacity than single CTCs do [234], [235]. Importantly, OSCC-derived CTCs often evade immune detection and resist anoikis, further contributing to their ability to metastasize [233].

CTCs are key indicators of oral cancer, and their continuous diagnosis in saliva samples of oral cancer patients makes them a promising molecule to be researched on. CTCs get disseminated from the primary lesions of the neoplasm when it acquires invasiveness. Although intact CTCs are not commonly found in saliva because physiological barriers limit their direct migration, tumor cell fragments and exosomes have been identified in salivary fluid [236], [237], [238]. These elements serve as reliable proxies for CTCs detection. For example, salivary markers such as miR-21, miR-31, and epithelial-specific antigens have been shown to be associated with CTC levels and overall tumor burden, suggesting their utility as complementary, non-invasive diagnostic indicators alongside blood-based liquid biopsies [233], [236], [237].

Detecting and analyzing CTCs in OSCC remains technically challenging owing to their low abundance, often fewer than 10 CTCs per 10 ml of blood, and their phenotypic diversity [233]. Current detection strategies are generally divided into labeled and unlabeled methods. Labeled techniques, such as the Food and Drug Administration (FDA)-approved CellSearch system, use immunomagnetic separation based on epithelial cell adhesion molecule (EpCAM) to isolate CTCs [238]. However, this approach may fail to capture CTCs that have undergone EMT and have lost EpCAM expression. To address this gap, newer systems incorporate broader antibody panels, including mesenchymal and stem cell markers, to increase detection coverage [233].

Unlabeled approaches leverage the unique physical traits of CTCs, such as size, deformability, and electrical properties, to isolate them without depending on specific surface marker antigens. Techniques such as microfluidic devices, density gradient centrifugation, and dielectrophoresis allow the enrichment of both epithelial and mesenchymal CTC populations [238]. Furthermore, innovations in scRNA sequencing and digital droplet PCR have enabled deeper molecular insights into individual CTCs, revealing details about their heterogeneity, resistance to treatment, and metastatic potential [233].

The clinical importance of CTCs in OSCC is gaining increasing recognition. Elevated CTC counts and the presence of cells with mesenchymal-like traits have been linked to poorer outcomes and more aggressive tumor phenotypes. Additionally, monitoring changes in CTCs levels during treatment can offer early indicators of therapeutic response or recurrence, often preceding visible changes on imaging. As such, incorporating CTCs-based assays into routine clinical workflows has the potential to significantly improve OSCC management, suggesting a minimally invasive avenue for personalized, dynamic cancer care [233].

### 10.4 Cytokines

Cytokine biomarkers in saliva have surfaced as promising tools for the early detection, disease prediction, and disease monitoring of OSCC. Cytokines are small signaling proteins released by immune and other cell types in response to various physiological and pathological triggers [239]. In OSCC, the TME is characterized by persistent inflammation and immune imbalance, which leads to distinct alterations in cytokine expression in body fluids, including saliva [125], [239]. Saliva being in closest proximity to the tumor site serves as pratical and non-invasive medium for the detection of oral cancer-promoting cytokines, which may have been from either systemic circulation or local tumor site or both [125].

A range of cytokines has been studied for their potential diagnostic relevance in OSCC. Notably, IL-6, IL-8, IL-1β and TNF-α are consistently upregulated in the saliva of OSCC patients compared with healthy individuals or those with non-malignant oral conditions [125]. Upregulated IL-6 levels in saliva have been linked with more advanced neoplasm stages and lymphatic node involvement, indicating its value in both diagnosis and prognosis [50], [239]. IL-8, a chemokine with strong inflammatory and angiogenic properties, is another cytokine that is frequently upregulated in OSCC. It supports cancer progression by attracting neutrophils, stimulating VEGF production, and inducing EMT, which enhances neoplastic invasion and metastasis [240]. While IL-1β, another key inflammatory molecule released by activated macrophages, contributes to the chronic inflammation characteristic of OSCC [240]. Another cytokine molecule, TNF-α, a major inflammatory mediator, has dual functions in OSCC. While it can trigger apoptosis in some contexts, chronic exposure within the tumor environment often leads to tumor-supportive behavior, largely by triggering the NF-κB signaling cascade [241]. Elevated TNF-α profiles in saliva have been linked with OSCC progression and are being explored as part of multianalyte panels to increase diagnostic precision [241], [242].

Thus, salivary cytokines offer not only a window into the biological activity of tumors but also a practical means for screening and follow-up [50], [239], [240], [241], [242], [243], [244], [245], [246], [247], [248], as highlighted in Table 6 [50], [239], [240], [241], [242], [243], [244], [245], [246], [247], [248]. These altered patterns reflect the inflammatory and immune milieu of the TME, and research continues to optimize their diagnostic and prognostic utility [125]. Incorporating cytokine profiling from saliva into clinical routines may greatly improve early detection and support more personalized approaches to OSCC care.

**Table 6:** Comparative expression of different cytokines in OSCC patients vs. healthy controls.

**Cytokines**	**Expression levels in healthy controls**	**Expression levels in OSCC**	**Expression in OSCC stages**	**Function in OSCC**	**References**
IL-6	Baseline/Low	3- to 5-fold upregulated in saliva/serum of OSCC patients compared to controls (*P*<0.001)	Stage I−II showed elevated expression; Stage III−IV showed markedly higher IL-6 expression	IL-6 promotes cellular division and activation of NLRP3-mediated inflammasome via JAK2/STAT3/Sox4 signal transduction pathway in OSCC-derived cells	[50], [239]
IL-8	Baseline/Low	4- to 6-fold upregulated in the saliva of OSCC patients compared to control patients (*P*<0.0001)	Stage I−II showed mildly elevated expression. Stage III−IV showed a significantly higher expression	IL-8 is a specific strong determinant of advanced-stage OSCC (stage III−IV)	[240]
IL-1β	Baseline/Low	Upregulated; 3- to 5-fold elevation with significant (*P*<0.01)	Very mildly elevated in early stage I−II stages, but strong elevation in later stage, i.e., III−IV	IL-1β is a specific strong determinant of advanced-stage OSCC (stage III−IV)	[240]
TNF-α	Baseline	Upregulated; 2- to 3-fold increase in OSCC saliva and serum samples as compared to controls (*P*<0.001)	Elevated expression is observed over all stages	Salivary TNF-α can be used as a prognostic biological marker of OSCC. Activates NF-κB pathway; promotes survival, inflammation, and tumor progression	[241], [242]
IL-10	Low expression	Upregulated by 2- to 4-fold compared to controls (*P*<0.05)	Slightly elevated in stage I−II, but strongly expressed in advanced stages	Elevated IL-10 expression has been closely linked to more aggressive clinical features and may serve as an independent predictor of survival outcomes, especially in patients diagnosed with early-stage OSCC	[243]
TGF-β1	Baseline	Upregulated by a 2-fold increase at the advanced OSCC stage (*P*<0.05)	A slight increase in stage I−II, but strong expression was observed in advanced stages	TGF-β influences the OSCC advancement through several interconnected mechanisms, notably by promoting EMT and enhancing angiogenesis, particularly during the advanced stages of the disease	[244]
IL-4	Baseline	Upregulated by 1.5- to 2-fold as compared to controls (*P*<0.05)	Elevated in every stage	Contributes to OSCC aggressiveness and poor survivability	[245]
IL-17	Low	Upregulated by almost a 3-fold increase in TSCC tissues (*P*< 0.01	Elevated in every stage	IL-17 is highly elevated in tongue SCC and is involved in the occurrence and development of TSCC, via JAK/STAT-signaling cascade	[246]
IL-12	Normal/High	Downregulated by almost 40%−50% in OSCC samples compared to controls (*P*<0.01)	Reduced as the stages advance; Low in stage I−II, but a higher reduction was observed in stage III−IV	IL-12 levels in the SCC group were substantially higher compared to those of healthy individuals	[247]
IFN-γ	Normal/High	Downregulated significantly (*P*<0.05) as compared to controls; No fold change reported	Reduction observed in every stage	IFN-γ contributes to immunosuppression in SCC by enhancing the expression profile of PD-L1 on small extracellular vesicles	[248]

NLRP3. NLR family pyrin domain containing 3; Sox4. SRY (sex-determining region Y) box 4; EMT. Epithelial-mesenchymal transition; TGF-β. Transforming growth factor β; JAK2. Janus kinase 2; STAT3. Signal transducer and activator of transcription 3; TNF-α. Tumor necrosis factor-α; IL. Interleukin; IFN-γ. Interferon γ; TSCC. Tongue squamous cell carcinoma; PD-L1. Programmed death-ligand 1; NF-κB. Nuclear factor κB

The comparative studies of cytokine expression in OSCC patients versus healthy controls highlighted the presence of a significantly dysregulated inflammatory microenvironment that evolves with disease progression. Cytokines, including IL-6, IL-8, IL-1β, TNF-α, and IL-17, are consistently elevated and further increase in advanced stages (III–IV). These cytokines demonstrate fold changes ranging from approximately 3 to 6 times greater than those found in healthy controls, which correlate with increased cellular proliferation, angiogenesis, and tumor invasiveness [50], [239], [240], [241], [242], [246]. These findings are consistent with the paradigm that a sustained proinflammatory environment contributes to malignant transformation and increases tumor aggressiveness.

In contrast, IL-10 and TGF-β1 play paradoxical roles as immune-regulatory cytokines in OSCC. Both cytokines are elevated even in the early stages, reflecting the early activation of immunosuppressive pathways that attenuate antitumor immunity [247]. Their persistence throughout various stages of the disease indicates a central role in facilitating immune evasion, and their strong association with poor prognosis underscores their potential as independent prognostic biomarkers [243], [244], [248]. However, the expression of these cytokines is less dependent on disease stage, limiting their utility for disease stratification [243], [244]. This situation contrasts with that of IL-12 and IFN-γ, which are progressively downregulated in OSCC patients relative to healthy individuals. The suppression of these cytokines underscores the gradual dismantling of Th1-type antitumor immunity and the establishment of a tumor-promoting immunosuppressive microenvironment [247], [248].

These cytokine expression patterns indicate that OSCC progression is characterized by a dual signature: the upregulation of proinflammatory mediators that promote tumor growth and invasion, combined with the suppression of immune-stimulating cytokines that would otherwise facilitate tumor clearance. This dynamic interplay reflects a complex immune-tumor interaction that is both stage-dependent and functionally significant. These findings provide a strong rationale for the development of multimarker cytokine panels rather than relying solely on single analytes, as such panels may more accurately capture the relationship between inflammation and immune suppression. However, challenges remain, including heterogeneity in sample sources (saliva versus serum), methodological variability, and the lack of standardized diagnostic thresholds, which currently impedes the translation of these findings into routine clinical practice. Future longitudinal studies that integrate cytokine profiling with genomic and metabolomic data are essential for validating these candidates as robust biomarkers for the diagnosis, prognosis, and treatment monitoring of OSCC.

### 10.5 Microbiomics

The salivary microbiome comprises a complex and extensive community of microbiota within the oral cavity that plays crucial roles in preserving oral cavity health and preventing disease. The scientific exploration of its composition, dynamics, and functional roles is termed “salivary microbiomics” [249], [250], [251]. Emerging evidence has increasingly linked alterations in the oral microbiome with the initiation and progression of OSCC. The oral environment harbors an array of bacteria, fungi, viruses, and archaea that engage in constant interactions with host tissues, the immune system, and with other bacteria [249], [252]. Disruption of this microbial balance, known as dysbiosis, has been recognized as a contributing factor in several cancers, including OSCC [249], [251], [252]. Studies have shown that the salivary microbiome in OSCC patients notably differs from that in healthy individuals, implying that certain microbial populations may facilitate carcinogenesis, promote tumor advancement, or help tumors evade immune detection [249], [250], [251], [252].

The salivary microbiome may influence OSCC development through mechanisms such as the modulation of immune responses, the promotion of genomic instability, and the induction of chronic inflammation. For example, bacteria such as *Fusobacterium nucleatum* have been shown to impair immune surveillance, thereby fostering tumor initiation [253]. Moreover, *Porphyromonas gingivalis* has been implicated in promoting genetic instability, encouraging mutations that drive cancer development [254]. Elevated detection of *Treponema denticola* has been linked with driving tumorigenesis in oral cancer cells by altering TGF-β signaling, leading to immune evasion and invasiveness [255], [256]. Further, *Enterococcus faecalis*, an oral and gut-associated bacterium often reported in higher amounts in OSCC tissues than in healthy samples. When this bacterium is co-cultured with oral cancer cells, the cells exhibited high proliferative activity, enhanced survival, and colony-forming activity by H_2_O_2_ production, which in turn upregulates EGF signaling and deregulates key apoptotic genes (p53, Bax), facilitating cancer cell growth, confirming their role in cancer progression. This bacterium’s genome consists of several virulence genes, such as gelatinase (*GelE*), *Asa*, and *Ace*, which help in its pathogenic interaction with neoplastic cells, leading to pro-tumorigenic [246]. While *Prevotella intermedia*, a periodontal pathogenic anaerobe, is often reported in oral microbiome analysis of OSCC patients more than any healthy controls, making it a potential OSCC microbiome biomarker. This bacterium exhibits pro-tumorigenic activity through immune evasion by activating the interferon-stimulated gene 15 (ISG15) axis. Upregulation of ISG15 leads to immune modulation, aberrant proliferation, and invasiveness characteristics in TME [257].

Furthermore, pathogenic bacteria often form biofilms that not only protect them from immune responses and therapeutic agents but also perpetuate chronic inflammation, thereby sustaining a tumor-promoting environment in OSCC [249], [250], [251]. Imbalanced microbial communities in the oral cavity can stimulate persistent inflammation, enhance cellular proliferation, support angiogenesis, and suppress apoptosis, all of which are central to tumor growth, as summarized in Table 7 [253], [254], [255], [256], [257].

**Table 7:** Relative abundance and detection frequency of the salivary microbiome in patients with oral squamous cell carcinoma (OSCC).

Microbial Species	Role in OSCC	Detection frequency in OSCC vs healthy controls	Results	References
*Fusobacterium nucleatum*	Tumor progression through modulation of protooncogenes	Detected in almost 60% – 80% OSCC samples and less than 30% in healthy controls	The interaction between *Fusobacterium nucleatum* and CDH1 was found to induce phosphorylation events that subsequently increased the expression profile of β-catenin. This activation led to the upregulation of cyclin D1 and Myc, ultimately promoting the proliferation of OSCC cells *via* the CDH1/β-catenin signaling pathway	[253]
*Porphyromonas gingivalis*	Signaling pathways alteration and induction of stemness	Detected in 50% – 70% of OSCC samples vs. <20% of controls	Induction of stemness by *Porphyromonas gingivalis* stemness OSCC cells *via* SCD1-mediated lipogenesis regulation	[254]
*Treponema denticola*	Alteration and activation of signaling pathways	Detected in approximately 25% – 40% OSCC samples, but controls report less than 10%	*Treponema denticola* could promote the OSCC initiation *via* the TGF-β pathway activation	[255]
*Enterococcus faecalis*	Activation of signaling pathways	25% – 30% samples confirm its presence, but controls show <5%	Activates EGFR through H_2_O_2_ production and promotes OSCC progression when co-cultured	[256]
*Prevotella intermedia*	Modulation of cytokine levels and key signal transducers	Detected in more than 50% of OSCC samples, while controls showed only a 15% frequency	It substantially enhanced tumor growth, invasion, angiogenesis, and metastatic potential, while also significantly altering the levels of inflammatory cytokines within the TME by activating the ISG15 axis	[257]

CDH1. Cadherin 1; SCD1. Stearoyl-CoA desaturase 1; TGF-β. Transforming growth factor β; EGFR. Epidermal growth factor receptor

### 10.6 Alterations in salivary protein levels post-OSCC treatment

The majority of OSCC patients undergo radiotherapy (RT), and very few studies have reported alterations in salivary biomarkers after treatment. Agurto *et al.* [258] performed a longitudinal study on 40 head and neck cancer patients who underwent intensity-modulated radiotherapy (IMRT) to examine their salivary protein profiles and oral mucositis (OM). More than half of the participants also received concurrent chemotherapy (cisplatin or carboplatin). Further, the patient’s saliva was collected before and after radiation treatment, which revealed that IMRT causes alterations in salivary profile and may lead to chronic impairment of saliva secretion. However, IMRT causes chronic alterations in saliva profile, but mucin levels (MUC7/5B) appeared high, which may be due to saliva volume, predicting that actual secretion did not change. While other key salivary molecules, such as α-amylase, albumin, and cystatin-S levels, decreased after treatment, partial recovery of amylase was reported over time. IgA profile remained constant after IMRT, which may serve as a useful biomarker for predicting OM severity in head and neck cancer patients.

A similar prospective longitudinal study, when conducted by Almhöjd *et al.* [259], led to similar findings, where survivors were deprived of salivary flow, in which levels of certain salivary molecules (MUC5B/MUC7) were altered due to low saliva volume secreted. Ramsay *et al.* [260] also reported similar findings; they utilized NMR-based saliva metabolomics to correlate findings with radio treatment, but they also found similar findings, highlighting the reduced saliva flow and its constituents upon treatment. All these longitudinal studies revealed that radiation therapy leads to less salivary flow, with certain constituents being overexpressed while other don’t.

## 11 Emerging trends in salivary diagnostics

Salivaomics refers to the holistic exploration of saliva, its components, and biological roles through the application of advanced omics technologies, such as salivary genomics, proteomics, transcriptomics, metabolomics, and microbiomics [261], [262], [263].

### 11.1 Integration of artificial intelligence and machine learning in salivaomics

The incorporation of salivaomics with AI/ML is playing a pivotal role in mitigating the burden of oral cancer by facilitating early, precise, and non-invasive diagnostic approaches. However, the high dimensionality and complexity of salivary multiomics data require sophisticated computational methods for reliable biomarker discovery and validation [264], [265].

AI and ML tools have emerged as essential in navigating this complexity. Different AI/ML algorithms, such as random forests (RFs), support vector machines (SVMs), and logistic regressions, have been effectively employed to analyze large-scale salivary datasets for classification, clustering, dimensionality reduction, and predictive modeling [232], [234]. These approaches excel at uncovering hidden patterns in the data and can discriminate between cancerous and healthy states with notable diagnostic accuracy in oral cancer prediction models [263], [264], [266]. The choice of AI methodology often depends on the task at hand: supervised learning techniques are commonly used for biomarker validation when outcome labels are available, whereas unsupervised learning supports exploratory analyses of unlabeled data. More recently, semi-supervised and self-supervised learning strategies have gained interest for their ability to harness both labeled and unlabeled data, thereby increasing model training efficiency and robustness [263], [267], [268]. Feature selection techniques such as least absolute shrinkage and selection operator (LASSO), minimum redundancy-maximum relevance (MRMR), and principal component analysis further refine these ML models by isolating the most informative biomarkers, improving their predictive performance, and minimizing overfitting [263], [269]. Importantly, AI-based frameworks can integrate salivary biomarkers with clinical, demographic, and imaging data, thereby enhancing diagnostic precision [270]. Several studies have already demonstrated the promise of AI-enhanced salivary biomarker platforms in detecting oral cancer, although larger, external validation cohorts are still needed to confirm these findings [268], [269].

AI has shown significant potential in identifying tumors within the oral cavity and areas of the oral and maxillofacial regions. However, the acceptance of its clinical application is still largely affected by ethical and regulatory concerns [269]. A key obstacle to implementing AI is the lack of dependable datasets, particularly salivary diagnostic datasets, which are currently insufficient [271]. Recent research has indicated that clinicians and bioinformaticians utilize tissue biopsy images (control, diseased, and undetected samples) to train artificial neural network (ANN) and convolutional (CNN) models. Following validation through receiver operating characteristic (ROC) and SVM techniques, these models may predict potential outcomes for patients with OSCC and the survival rates of patients [269], [270]. Tseng *et al.* [272] utilized an AI-integrated case-control design to analyze autoantibody profiles in saliva samples of both OSCC and healthy individuals. They utilized different ML models, such as logistic regression, random forest, and SVM, to develop OSCC risk prediction models by integrating saliva-derived autoantibody data and demographical elements. Through this, the researchers achieved the best AUC=0.80, supporting the feasibility of integrating salivary biomarkers with clinical findings to distinguish between tumor, non-tumor, and invasive samples. This study also highlighted that incorporating multiple ML models with salivary autoantibodies data surpassed the performance of individual ML models.

Adeoye *et al.* [273] utilized OSCC and OPMDs saliva samples to develop a nested cohort, where they tried to analyze DNA methylation biomarkers through the reduced representation bisulfite sequencing (RRBS) technique. Further, he incorporated multiple ML models to pinpoint genome-wide DNA methylation biomarkers. Among 8 of the ML models utilized, the linear SVM model based on 11 LASSO-selected DMRs achieved optimal results, with AUC=1.00, sensitivity=1.00, specificity=1.00, calibration=1.00, in distinguishing OSCC from OPMDs. Overall, these findings indicated that analyzing salivary methylomes integrated with machine learning provides a highly precise, non-invasive diagnostic tool for oral cancer diagnosis [273].

**Fig. 6:**
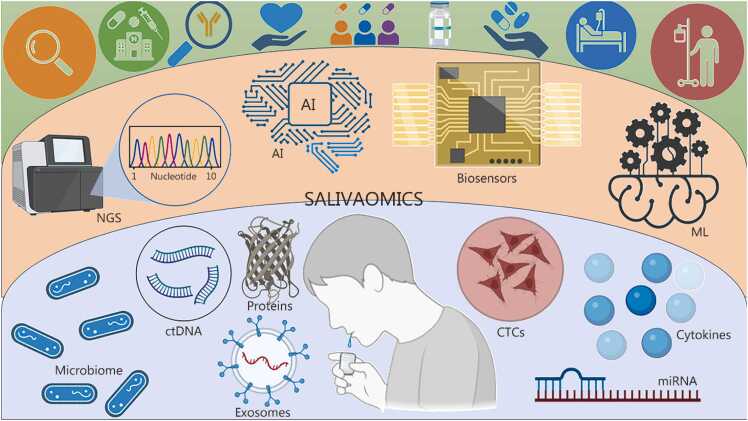
Salivaomics integrating multi-omics, biosensors, and AI/ML-driven precision diagnostics in oral cancer detection. This figure represents an emerging concept of “Salivaomics”, outlining the salivary biomarker-based research in conjunction with modern AI/ML tools, which can lead to quick detection and prognosis with improved therapeutic outcomes. Saliva is a non-invasive diagnostic medium containing diverse biomolecules (represented in the blue region) which if exploited using advanced techniques and integrated into diagnostic models through AI and ML (represented in light peach colored region) can lead to prompt diagnosis, limit hospitalization, timely palliative care, precise, and personalized medicine, easy monitoring, and enhance drug delivery (represented in green region). ctDNA. Circulating tumor deoxyribonucleic acid; CTCs. Circulating tumor cells; miRNA. Micro ribonucleic acid; NGS. Next-generation system; AI. Artificial intelligence; ML. Machine learning.

Ultimately, this integrated approach could redefine saliva as a central tool in cancer diagnostics, enabling timely intervention and more effective management of oral cancer, as shown in Fig. 6. The long-term success of this strategy hinges on ongoing research, rigorous validation, and the development of clinically applicable AI models with proven efficacy in real-world settings [266], [271].

### 11.2 Electric field-induced release and measurement and droplet digital PCR: a novel platform for non-invasive molecular diagnostics

One such innovative platform is EFIRM, an electrochemical method designed for rapid and non-invasive detection of molecular biomarkers directly from biofluids, eliminating the need for complex extraction or amplification steps [274], [275]. EFIRM’s high sensitivity stems from the use of engineered nucleic acid probes tailored to recognize low-abundance targets, enhanced electrode surfaces for improved probe loading and interaction, and optimized electric waveforms that promote efficient and selective hybridization [274], [276], [277]. In cancer diagnostics, EFIRM has proven effective in identifying critical mutations such as EGFR variants in both saliva and plasma [278], [279]. The method involves anchoring capture probes onto gold electrodes *via* electro-polymerization and then applying cyclic square-wave electric fields that drive target DNA toward the probe surface while minimizing non-specific binding. This dynamic field ensures precision by favoring perfect matches and reducing background noise [274], [275], [276]. EFIRM has demonstrated the ability to detect single-nucleotide variants from small sample volumes in less than 30 min, making it a powerful tool for rapid liquid biopsy. Its clinical value was first highlighted by the successful diagnosis of EGFR mutations in salivary ctDNA from non-small cell lung cancer (NSCLC) patients [274], [275], [276]. Subsequent research has shown its effectiveness in monitoring therapeutic responses and predicting disease recurrence, often surpassing conventional techniques such as dPCR and gene sequencing [262], [279].

However, ddPCR provides significant advantages over EFIRM in the detection of salivary ctDNA, especially in terms of sensitivity and reliability [280]. ddPCR is a highly precise methodology that partitions samples into minute droplets, allowing for the measurement of light emitted after the assay. This technique is capable of accurately quantifying a wide range of concentrations, from extremely low to significantly high levels. It exhibits robust performance even in the presence of potential interferences from various substances found in saliva. The significance of this capability is underscored by the complex composition of saliva, which may contain diverse constituents that could impact the test results [280], [281]. Validated ddPCR assays reliably reach a limit of detection (LOD) of approximately 0.01% VAF, which corresponds to the identification of approximately one mutant molecule in 10,000 wild-type molecules [281], [282]. Additionally, in HPV ctDNA tests, LODs as low as 0.04 copies/ml have been observed in plasma, significantly surpassing traditional PCR techniques [280], [281], [282]. In contrast, EFIRM might feel appealing owing to its quick testing capabilities without amplification on small sample sizes, but it suffers from limited interlaboratory calibration and is prone to interference from proteins and microbiota. EFIRM’s LOD shows significant variability and is dependent on the target: specific EGFR mutations can be identified at single-digit copy numbers, whereas exon 19 deletions necessitate close to 5000 copies for consistent detection, highlighting sensitivity to probe design and the effects of the salivary matrix [277], [283].

In clinical settings, ddPCR has demonstrated extensive multicenter validation in various cancers. Specifically, in HPV-positive head and neck cancer, the detection of ctDNA in both saliva and plasma via ddPCR achieves a sensitivity of approximately 70%, in contrast to the sensitivity of only 20.6% for qPCR. This substantial difference supports the adoption of ddPCR for the monitoring of minimal residual disease [280]. EFIRM, in contrast, has demonstrated encouraging yet somewhat limited pilot data, particularly in the detection of EGFR mutations in non-small cell lung cancer, yielding AUCs between 0.90 and 0.94, alongside a remarkable 100% sensitivity observed in very small cohorts. However, large-scale validation specific to saliva in the context of OSCC is lacking [277], [283].

With respect to costs and scalability, ddPCR necessitates the use of specialized equipment, including droplet generators, fluorescence readers, and specific consumables, which culminate in relatively elevated per-test costs. Furthermore, additional expenditures arise from the procurement of DNA extraction kits and the requirement for trained personnel [284]. In contrast, EFIRM is engineered to function as a true point of care solution for early OSCC detection as it has a rapid turnaround time, easy portability, and utilizes minimal volumes of saliva or plasma with no extraction or amplification processes required [273], [274], [275], [276], [277], [283]. Preliminary pilot studies suggest that EFIRM could be implemented at a lower marginal cost once scaled, particularly within point-of-care environments. However, commercial EFIRM platforms remain in the developmental stage; their scalability is currently constrained by the lack of standardized commercial kits, issues of reproducibility across different platforms, the necessity for multicenter clinical validation, and current cost estimates are projections rather than validated market figures. ddPCR is a well-established technique, with commercial platforms from companies such as Bio-Rad and Thermo Fisher already widely deployed in research and clinical laboratories [283]. Moreover, despite being less validated, EFIRM holds promise as a cost-effective, scalable, and rapid diagnostic platform, particularly for saliva-based OSCC screening, pending advancements in multicenter validation and commercial standardization [283], [285].

While ddPCR is regarded as the gold standard for ctDNA quantification due to its validated methodology, consistency, and established market presence. The scalability of ddPCR is moderate; although the method is robust, its throughput is limited to 96–384 samples per run, with batch processing times ranging from 4 – 6 h. Consequently, ddPCR is best suited for centralized laboratories rather than for rapid, bedside testing scenarios, limiting its scalability for mass OSCC screening [282].

### 11.3 Saliva exosomics and its diagnostic implications

Saliva exosomics, a budding subfield within salivaomics, focuses on investigating the molecular contents of small (30 – 100 nm), membrane-enclosed EVs released by most cell types through the endosomal pathway [262]. These vesicles circulate in various bodily fluids, such as saliva, blood, and CSF, and play essential roles in cell-to-cell communication by transporting biologically active molecules that modulate cellular signaling pathways and maintain physiological balance [286]. As a unique and distinct component of saliva, exosomes provide a relatively stable and uncontaminated source of biomarkers that are protected from enzymatic degradation by their lipid bilayer. This inherent stability, along with their diverse molecular cargo, positions exosomes as promising candidates for diagnostic use [286].

Because of their established role in cancer biology, salivary exosomes are particularly well-suited for identifying tumor-associated biomarkers that reflect cancer initiation, progression, and response to therapy. Continued research into the exchanges between tumor cells and salivary exosomes has the potential to increase our knowledge of pathogenicity and accelerate the advancement of non-invasive diagnostic tools [262], [287].

### 11.4 Ultrashort cfDNA in saliva: a frontier in non-invasive diagnostics

uscfDNA is a recently identified subtype of ccfDNA distinguished by its fragment length ranging from 25 to 75 nt, with a peak at approximately 50 nt, considerably shorter than the conventional 167 base pair mononucleosomal cfDNA (mncfDNA) [288]. This advancement became possible through the integration of specialized extraction techniques optimized for short DNA fragments and single-stranded library preparation methods, which collectively enhance the detection of sequences typically overlooked by standard sequencing approaches.

Interestingly, uscfDNA appears to be more prevalent in healthy individuals than in those with cancer and is thought to originate from regions of open chromatin, while functional analyses indicated that uscfDNA and mncfDNA differed in their genomic distributions, particularly colocalized in areas such as introns, intergenic sequences, and gene promoters [289], [290], [291]. The unique properties of uscfDNA, especially its shorter fragment size, make it a suitable candidate for detection through EFIRM technology, potentially increasing the sensitivity and efficiency of liquid biopsy applications, even with very small sample volumes [277], [288]. Although it was initially identified in plasma, the detection of uscfDNA in saliva highlights its broader potential for non-invasive diagnostic applications [285], [286], [287]. Continued investigations into the biological sources and therapeutic impact of uscfDNA are expected to further clarify its value in early disease detection and its broader relevance in precision medicine [262], [284].

### 11.5 Promising salivary biomarkers for future clinical use

The clinical evidence supporting salivary biomarkers in OSCC has progressed from initial discovery cohorts to multisite validation and evaluations of diagnostic devices; however, significant gaps remain. Extensive proteomic and transcriptomic studies, such as those conducted by Yu *et al.* [290], have demonstrated that multianalyte saliva panels can achieve clinically relevant accuracy, with one four-protein panel reporting a sensitivity of 87.5% and a specificity of 80.5%. Prospective community cohort studies, including those evaluating CD44/total protein oral rinses, further indicate the feasibility of longitudinal surveillance and early detection in high-risk populations [291]. Nonetheless, many device trials and observational studies report heterogeneous metrics, with variability in endpoints, sampling procedures, and analytical methodologies, which limits direct comparison [192], [193], [194]. Reproducibility remains satisfactory for well-standardized ELISA and qPCR assays, but declines in exploratory platforms such as LC-MS and untargeted metabolomics unless centralized quality control is implemented [192], [193], [194], [197]. Interpatient variability driven by oral hygiene, smoking, inflammation, dietary influences, and microbiome differences represents a major confounder, reducing specificity in the general population; many promising biomarkers perform optimally only in enriched high-risk cohorts [195]. Furthermore, several registered diagnostic device studies, including OncAlert and OncAlert RAPID (NCT03239834), are ongoing [https://clinicaltrials.gov/search?term=NCT03239834]; however, comprehensive data on diagnostic performance and multicenter reproducibility remain limited in public registries. Consequently, broader clinical adoption will require multicenter randomized trials or large-scale prospective validation studies guided by harmonized standard operating procedures.

Several salivary biomarkers have been identified with current diagnostic methods, but very few are feasible for clinical use [292], [293]. The diagnostic performance of several promising salivary biomarkers that have been evaluated in OSCC patients is summarized in Table 8 [294], [295], [296], [297], [298], [299], [300], [301], [302], [303], [304]. Among inflammatory markers, IL-8 and IL-1β are consistently elevated in OSCC and premalignant lesions, with IL-8 demonstrating strong reproducibility across multiple studies (sensitivity 86%–89%, specificity 85%–90%, AUC=0.93) [292], [293], while IL-1β also shows potential diagnostic value (AUC=0.82) [296]. CD44, a soluble adhesion molecule, is particularly noteworthy as the only biomarker to receive FDA approval for adjunctive OSCC testing [297], underscoring its translational relevance. Salivary miRNAs, particularly miR-21 [296], [297] and miR-184 [299], display encouraging accuracy; however, their performance may vary depending on the tumor subsite, with miR-184 showing greater specificity for tongue SCC.

**Table 8:** Promising salivary biomarkers for OSCC detection.

**Promising salivary biomarkers**	**Sensitivity (%)**	**Specificity (%)**	**AUC**	**Diagnostic utility**	**References**
IL-8	86–89	85–90	0.93	Widely validated; key inflammatory marker in OSCC	[294], [295]
IL-1β	78	80	0.82	Elevated in premalignant and malignant lesions	[296]
CD44	79	84	0.86	FDA-approved for adjunctive OSCC testing	[297]
miR-21	82	81	0.88	Oncogenic miRNA: highly stable in saliva	[298], [299]
miR-184	82	76	0.83	Reported specificity for tongue SCC	[300]
CYFRA 21-1	77	83	0.81	Reflects epithelial turnover	[301]
HPV DNA (ctDNA in saliva)	91	90	0.92	Strong diagnostic accuracy for HPV+ oropharyngeal cancers	[302]
p53 autoantibodies	72	78	0.79	Detects tumor-associated immune response	[303]
*EGFR* mRNA	80	83	0.87	Consistently upregulated in OSCC	[304]

IL. Interleukin; CD44. Cluster of differentiation 44; miR-21. microRNA 21; CYFRA. Cytokeratin fragment; HPV. Human papillomavirus; EGFR. Epidermal growth factor receptor; FDA. Food and Drug Administration; OSCC. Oral squamous cell carcinoma; SCC. Squamous cell carcinoma; AUC. Area under the curve

Protein and nucleic acid biomarkers demonstrate diagnostic promise. CYFRA 21-1, which reflects epithelial turnover, achieves an AUC of 0.81 [301], whereas *EGFR* mRNA has been consistently reported to be upregulated in OSCC, with good accuracy (AUC=0.87) [304]. Viral and immune-associated markers broaden the biomarker spectrum: detection of ctDNA achieves the highest diagnostic accuracy (sensitivity=91%, specificity=90%, AUC=0.92) in HPV-positive oropharyngeal cancers [302], whereas p53 autoantibodies provide moderate sensitivity and specificity (AUC=0.79) [303]. Nonetheless, several limitations remain. Reported performance varies across studies owing to heterogeneity in study design, patient cohorts, saliva collection protocols, and analytical methodologies. Interpatient variability, arising from oral hygiene, inflammation, smoking, diet, and microbiome differences, further reduces specificity in the general population. These challenges emphasize the need for large-scale, multicenter validation studies, harmonized methodologies, and the integration of multianalyte panels to improve reproducibility and achieve clinical utility.

## 12 Comparative evaluation of salivary biomarkers and established diagnostic standards in OSCC

Current diagnostic standards for OSCC primarily rely on visual and tactile examinations, followed by histopathological biopsy. Additionally, adjunctive screening methods, including toluidine blue staining, brush cytology, and autofluorescence, demonstrate variable accuracy that is contingent upon the clinician’s level of expertise and the specific characteristics of the lesions being assessed. Salivary diagnostics have emerged as a compelling alternative. Salivary biomarkers have the potential to outperform existing standards, particularly compared with traditional serum and plasma biomarker platforms.

### 12.1 Diagnostic accuracy: saliva vs. current standards

Evidence from meta-analyses indicates that various salivary biomarkers demonstrate diagnostic capabilities that are at least as good as, and in some instances better than, adjunctive diagnostic tools [195], [295], [305]. A network meta-analysis consolidating several systematic reviews identified MMP-9 and chemerin as the highest-performing individual markers, achieving sensitivities of up to 0.94 and balanced accuracies of approximately 0.93, which are significantly greater than the usual sensitivity of 70%–80% associated with toluidine blue or autofluorescence [305]. A dedicated meta-analysis examining IL-8, CYFRA21-1, and CD44 revealed pooled AUCs of 0.88, 0.90, and 0.91, respectively, whereas a biomarker panel reached an AUC of approximately 0.92 (sensitivity=88%, specificity=90%) [306]. In contrast, adjunctive methods typically do not surpass 85% in terms of sensitivity and specificity in practical applications. These aggregated values underscore the possibility that salivary panels may statistically outperform existing chairside adjuncts. Nevertheless, unlike biopsy, which is conclusive, salivary biomarkers have yet to be validated in large prospective cohorts to establish clinical superiority in real-world settings [195], [306].

### 12.2 Saliva vs. serum and plasma biomarkers

Serum and plasma have traditionally been the preferred biofluids for developing biomarkers, as they offer a stable matrix and have been extensively validated in cancer research (e.g., CYFRA21-1 for lung cancer) [307]. However, in OSCC, analytes from saliva often demonstrate greater discriminative accuracy than their plasma counterparts do, especially for locally secreted cytokines and epithelial markers. For example, the salivary AUC of IL-6, IL-8 exceeds 0.85, whereas the plasma AUC falls within the 0.70—0.80 range [308]. Saliva provides a more accurate representation of interactions within the local TME because tumor DNA, RNA, and proteins are directly released into oral fluids [309]. Nonetheless, plasma biomarkers may exhibit greater resilience to systemic confounding variables, whereas salivary biomarkers are more prone to the effects of oral inflammation and periodontal disease, which can compromise their specificity. Consequently, the primary advantage of saliva lies in its closer association with tumor biology, whereas plasma offers increased systemic stability [310].

### 12.3 Methodological and technological considerations in saliva vs. serum

The performance of diagnostic platforms necessitates the methodological and technological consideration of the biological framework, as this directly affects the sensitivity and specificity. The technologies utilized for the analysis of saliva have undergone rapid advancements. Preliminary studies have relied predominantly on ELISA-based quantification of individual cytokines, such as IL-6, IL-8, and TNF-α, which have resulted in inconsistent findings due to variability in assay methods and limited sample sizes [202]. Recent progress in proteomics, metabolomics, transcriptomics, and next-generation sequencing has enabled the development of multimarker discovery panels that demonstrate increased reproducibility. For example, salivary metabolomics employing NMR and LC-MS has identified specific discriminatory metabolites, including taurine, choline, and valine, which exhibit reported AUCs ranging from 0.85—0.91 [311]. Similarly, the profiling of salivary microRNAs, specifically miR-31, miR-125a, and the miR-200 family, has demonstrated significant diagnostic potential, with sensitivities exceeding 80% in small cohorts [312].

In contrast, serum biomarker research benefits from established standardization protocols and validated assay methodologies. Plasma CYFRA21-1, SCC antigen, and p53 autoantibodies can be routinely quantified *via* clinical-grade assays, whereas salivary biomarker assays frequently lack FDA-approved platforms and are significantly affected by preanalytical variability. Numerous studies consistently emphasize that variations in saliva collection methods, whether stimulated or unstimulated, centrifugation techniques, storage conditions, and normalization strategies, result in substantial discrepancies among studies. Without harmonized protocols, the reproducibility of results across different laboratories remains inadequate, thereby undermining assertions of superior performance [277], [309].

### 12.4 Cohort design and clinical relevance

Comparative diagnostic performance is strongly influenced by study design and cohort composition for better interpretability. The majority of research on salivary biomarkers conducted thus far employs a case-control approach, contrasting established OSCC patients with healthy subjects [295], [305]. Such study designs tend to inflate diagnostic performance metrics by maximizing the signal-to-noise ratio, whereas genuine screening cohorts should encompass OPMDs and benign inflammatory diseases. When controls include patients with OPMDs or periodontitis, the specificity of cytokine-based indicators such as IL-6 and IL-8 significantly decreases, indicating their vulnerability to interference from oral inflammatory factors [310]. Studies involving serum and plasma typically involve larger participant cohorts and utilize prospective collection methods, which increase the generalizability of the findings. Importantly, only a limited number of studies on salivary biomarkers have included more than 200 participants [305], whereas the majority of meta-analyses involve small studies with fewer than 100 participants, making them vulnerable to the effects of small-study bias [295], [303].

### 12.5 Interpatient variability and confounding effects in saliva compared to plasma biomarkers

Interpatient variability is a major determinant of the performance of the diagnostic platforms, and hence, it necessitates the evaluation of the reported accuracy metrics. A prevalent critique pinpointed in numerous studies is that variability among patients restricts the clinical applicability of saliva-based diagnostics [195]. The observed variability elucidates the inconsistencies in the accuracy of biomarkers such as IL-1β, TNF-α, and lactate dehydrogenase (LDH) across various cohorts. While the utilization of multimarker panels has the potential to reduce noise, the challenge of reproducibility remains a significant obstacle. Plasma biomarkers are less subject to the confounding effects of oral environmental factors; however, systemic inflammation continues to play a role. Therefore, although saliva exhibits heightened sensitivity due to its proximity, its specificity is adversely affected by patient-level confounders in comparison to serum and plasma [6], [195].

### 12.6 Clinical outcomes beyond diagnostic accuracy: existing evidence and research gaps

While salivary panels have AUCs greater than 0.90, few studies have evaluated results beyond cross-sectional diagnostic accuracy. To achieve genuine clinical superiority, it is essential to demonstrate earlier detection, minimal time to biopsy, or enhanced survival rates. To date, no salivary biomarker study has prospectively shown these endpoints [1], [5]. Serum and plasma markers have not been substituted for histology, but they have demonstrated effectiveness in prognosis, treatment monitoring, and surveillance of recurrence in other cancer types. To apply saliva for such purposes, long-term cohorts will be needed [195].

Overall, salivary biomarkers exhibit greater overall diagnostic accuracy than adjunctive tools such as toluidine blue and autofluorescence. In certain situations, these methods outperform serum or plasma tests for detecting localized tumors. Nevertheless, a biopsy is still essential because of its definitive nature [195], [295]. The advantages of saliva include its closeness to tumor biology, simplicity in collection, and potential for multiomics discovery, whereas its disadvantages include preanalytical variability, vulnerability to local inflammatory interference, and the absence of clinical-grade assays. Plasma and serum offer systemic reliability and established standardization, but they often lack the localized sensitivity that saliva provides [195], [295], [305].

For salivary biomarkers to claim genuine “outperformance”, they must present prospective, reproducible accuracy in large cohorts involving OPMDs and benign oral conditions, as well as standardized collection and analytical methods. Saliva should be viewed as a promising complementary platform with the potential to match or exceed adjunctive techniques, but is not yet a substitute for histopathological verification [295].

## 13 Translational benefits, existing challenges, and future directions in salivary biomarker research

**Fig. 7:**
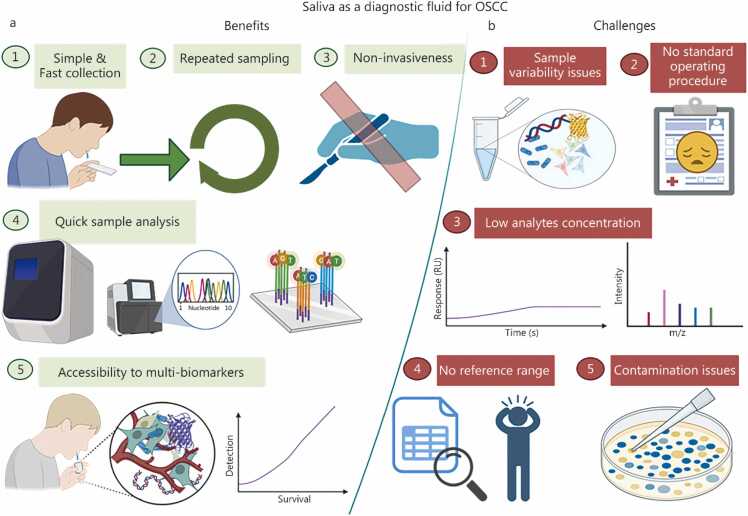
Benefits (**a**) and challenges (**b**) of salivary diagnostics for oral squamous cell carcinoma (OSCC). Salivary diagnostics offer simple & fast sample collection, repeated non-invasive sampling (through saliva collection), cost-effective, prompt, pain-free OSCC diagnosis with access to multi-panel biomarkers, but variability in patient samples, lack of standardization, low analyte concentration with no reference range, and contamination issuespresent translational challenges at preliminary levels.

Saliva can be the most promising liquid for early non-invasive diagnosis and OSCC detection because of its proximity to the OSCC TME. Research into salivary biomarkers for OSCC presents numerous advantages, especially the non-invasive and convenient nature of saliva acquisition. This method is low-stress, cost-efficient, and amenable to repeated sampling, making it particularly valuable for routine screening and monitoring, especially in individuals at elevated risk [278]. Nevertheless, salivary biomarkers also hold several limitations, such as the fact that the concentrations of some biomarkers in saliva are often lower than those found in tissue or blood, which may compromise detection sensitivity. Additionally, the compositional heterogeneity of saliva is also affected by variables such as dietary habits, oral hygiene practices, lack of standardized protocol, and overall systemic health, which can lead to inconsistent results and pose challenges to standardization [278], as shown in Fig. 7**.**

Large-scale clinical trials are essential to confirm their diagnostic and prognostic ability, which will allow saliva-based diagnostic methods to overcome analytical and clinical hurdles through a strong commitment to methodological rigor and validation [277], [278], [310]. To ensure successful clinical translation, it is necessary to implement standardized protocols for saliva acquisition, processing, data interpretation, and AI/ML integration, which will allow for reproducibility, prompt detection, accurate OSCC diagnosis, precise tumor classification with enhanced clinical outcomes [263].

Ongoing and future investigations will further explore the role of salivary biomarkers in tracking disease progression, disease recurrence, and therapeutic efficacy, positioning saliva as a valuable, non-invasive tool in the broader context of personalized medicine.

## 14 Conclusions

Salivary biomarkers may serve as frontiers in the early detection and monitoring OSCC, offering non-invasive, pain-free diagnoses compared to the traditional ones. Although saliva serves exclusively as a diagnostic medium and not as a route of cancer metastasis, which occurs through the bloodstream and lymphatic system. Various proteins, nucleic acids, metabolites, and exosomal components have demonstrated favorable sensitivity, specificity, and area under the curve values in differentiating OSCC patients from healthy individuals or those with potentially malignant conditions. Since the majority of the evidence is derived from a case study on a limited population, it overwhelms the diagnostic performance relative to worldwide population screening. Furthermore, the impact of confounding oral conditions, such as periodontal disease, smoking, and microbial dysbiosis, on biomarker specificity is not yet fully understood. Only a number of biomarkers came in the spotlight for their clinical use. Additionally, there is a notable absence of outcome-based studies highlighting a significant impact on earlier diagnosis or improved survival.

In conclusion, while research on salivary biomarkers for the diagnosis of OSCC is an exciting and rapidly advancing field, successful clinical translation will depend on the standardization of methodologies, rigorous validation across diverse populations, integration into multiplex panels, and clear evidence of tangible clinical benefits. Addressing these gaps will be essential for moving saliva-based diagnostics from experimental promise to practical application, thereby improving outcomes for patients with OSCC.

## Abbreviations

**Table:** 

2-DG	2-deoxy-D-glucose
2D-GE	Two-dimensional gel electrophoresis
3’UTRs	3’ untranslated regions
AI	Artificial intelligence
Akt	protein kinase B
ANX4	Annexin A4
AREG	Exosomal amphiregulin
AUC	Area under the curve
Bcl-6	B-cell lymphoma 6
BQ	Betel-quid
C/EBPβ	CCAAT/enhancer binding protein β
CAFs	Cancer-associated fibroblasts
CD	Cluster of differentiation
cfDNA	Cell-free DNA
CFH	Complement factor H
CLEC3B	C-Type lectin domain family 3 member B
CNN	Convolutional neural network
CRP	C-reactive protein
CSCs	Cancer stem cells
CTCs	Circulating tumor cells
ctDNA	Circulating tumor DNA
CTLA-4	Cytotoxic T-lymphocyte antigen-4
CXCL12	C-X-C chemokine ligand 12
CXCR4	C-X-C chemokine receptor 4
ddPCR	Droplet digital PCR
DMR	Differentially methylated region
DNMTs	DNA methyltransferases
DR	Diffusion reflection
DUSP1	Dual-specificity phosphatase 1
E2F	Early region 2 binding factor
ECM	Extracellular matrix
EEs	Early endosomes
EGF	Epidermal growth factor
EGFR	Epidermal growth factor receptor
EHF	Epithelial height transcription factor
ELISA	Enzyme-linked immunosorbent assay
EMT	Epithelial-mesenchymal transition
epCAM	Epithelial cell adhesion molecule
ESCRT	Endosomal sorting complex required for transport
ESS	Elastic scattering spectroscopy
EV	Extracellular vesicles
EZH2	Enhancer of zeste homolog 2
FAD	Flavin adenine dinucleotide,
FAO	Fatty acid oxidation
FDA	Food and Drug Administration
FGA	Fibrinogen alpha chain
FGF	Fibroblast growth factor
FISH	Fluorescent in situ hybridization
FNAC	Fine needle aspiration cytology
FOXO	Forkhead box, class O
GAPDH	Glyceraldehyde-3-phosphate dehydrogenase
GET4	Guided entry of tail-anchored proteins 4
GNPs	Gold-based nanoparticles
GNRs	Gold nanorods
GPCRs	G protein-coupled receptor
GSK	Glycogen synthase kinase
HA3	Histatin 3
HGF	Hepatocyte growth factor
HMT	Histone methyl-transferase
HMTi	Histone methyl-transferase inhibitor
HNC	Head and neck cancer
HNSCC	Head and neck squamous cell carcinoma
HPV	Human papillomavirus
Hsp27	Heat factor H
IARC	International agency for research on cancer
IHC	Immunohistochemistry
IL	Interleukin
ILVs	Intraluminal vesicles
IMRT	Intensity-modulated radiotherapy
ISG15	Interferon-stimulated gene 15
Ki-67	Kiel-67
LASSO	Least absolute shrinkage and selection operator
LC-MS	Liquid chromatography-mass spectrometry
LDH	Lactate dehydrogenase
let	Lethal
LINE-1	Long interspersed nuclear element 1
LOD	Limit of detection
LOH	Loss of heterozygosity
M2BP	Mac2 binding protein
MAPK	Mitogen-activated protein kinase
MAPKKK	MAPK kinase kinase
MAPSs	Molecularly activated plasmonic nanosensors
MDM2	Murine double minute 2 homolog
MDSCs	Myeloid-derived suppressor cells
miRNAs	MicroRNAs
ML	Machine learning
MMP	Matrix metalloproteinases
mncfDNA	Mononucleosomal cfDNA
MRP14	Myeloid-related protein 14
MS	Mass spectrometry
mTOR	Mammalian target of rapamycin
mTORC2	Mechanistic target of rapamycin complex 2
MUC5B/7	Mucin 5B/7
MVBs	Multivesicular bodies
NADH	Nicotinamide adenine dinucleotide
NF-κB	Nuclear factor κB
NICD	Notch intracellular domain
NMR	Nuclear magnetic resonance
NNMT	Nicotinamide N-methyltransferase
NSCLC	Non-small cell lung carcinoma
OAZ1	Ornithine decarboxylase antizyme 1
OLP	Oral leukoplakia
OM	Oral mucositis
OPMD	Oral potentially malignant disorder
OSCC	Oral squamous cell carcinoma
OSF	Oral submucous fibrosis
PCR	Polymerase chain reaction
PD-1	Programmed cell death protein 1
PDCD4	Programmed cell death protein 4
PDK1	Phosphoinositide-dependent kinase-1
PD-L1	Programmed death-ligand 1
PI3K	Phosphoinositide 3 kinase
PIP2	Phosphatidylinositol 4,5-bisphosphate
PIP3	Phosphatidylinositol 3,4,5-trisphosphate
PRC2	Polycomb repressive complex 2
PTEN	Phosphatase and tensin homolog
qPCR	Quantitative polymerase chain reaction
RFs	Random forests
RNF4	Ring finger protein 4
RNF114	Ring finger protein 114
ROC	Receiver operating characteristic
RRBS	Reduced representation bisulfite sequencing
RT	Radiotherapy
RTKs	Receptor tyrosine kinase
RT-PCR	Reverse transcription polymerase chain reaction
SAM	S-adenosylmethionine
SAT	Spermidine/spermine N1-acetyltransferase
SCC	Squamous cell carcinoma
SCC1	Sister chromatid cohesion protein 1
SDF-1	Stromal-derived factor-1
SERPINA1	Serpin family A member 1
SETD2	SET domain containing 2
SFRF2	Secreted frizzled-related protein 2
SIRT1	Silent information regulator sirtuin 1
SLNB	Sentinel lymph node biopsy
SMAD2	Suppressor of mothers against decapentaplegic homolog 2
SMAD3	Suppressor of mothers against decapentaplegic homolog 3
SMAD4	Suppressor of mothers against decapentaplegic homolog 4
SOCS1	Suppressor of cytokine signaling 1
sPA	Spectroscopic photoacoustic
SPR	Surface plasmon resonance
STAT3	Signal transducer and activator of transcription 3
SUV39H1	Suppressor of variegation 3-9 homolog 1
SVM	Support vector machine
TAMs	Tumor-associated macrophages
TAZ	Transcriptional co-activator with PDZ-binding motif
TGF	Transforming growth factor
TGF-β	Transforming growth factor β
Th1	T-helper 1
THOP1	Thimet oligopeptidase 1
TIMP1	Tissue inhibitor of metalloproteinase 1
TME	Tumor microenvironment
TNF	Tumor necrosis factor
TNM	Tumor node metastasis
TP53	Tumor protein 53
TPC	Total protein concentration
TPS	Total protein secretion rate
TSC2	Tuberous Sclerosis Complex 2
TSGs	Tumor suppressor genes
TβRI/II	TGF-β receptor type I
uscfDNA	Ultrashort cell-free DNA
UTRS	Untranslated regions
VAF	Variant allele frequency
VEGF-A	Vascular endothelial growth factor A
YAP	Yes-associated protein

## Ethics approval and consent to participate

Not applicable.

## Authors’ contributions

The experimentation, method development, and manuscript drafting were carried out by AP, while TKU and FA contributed to the study’s conceptualization, supervision, manuscript review, editing, and resource management. AA, HS, and MS were involved in critical manuscript revision and editing. All authors read and approved the final manuscript.

## Funding

This work was supported by the Brown Cancer Center, CCII CoBRE Grant (P20GM135004), and the Jewish Heritage Fund for Excellence Research Enhancement Grant Program (G7043).

## Data Availability

Not applicable.
